# Environmental disturbances of trophic interactions and their impacts on a multihost sapronotic pathogen

**DOI:** 10.1093/femsec/fiag006

**Published:** 2026-01-28

**Authors:** Ahmadou Sylla, Christine Chevillon, Magdalene Dogbe, Kayla M Fast, Jennifer L Pechal, Alex Rakestraw, Matthew E Scott, Michael W Sandel, Heather Jordan, Mark Eric Benbow, Jean-François Guégan

**Affiliations:** MIVEGEC, Université de Montpellier (UM), Centre National de la Recherche Scientifique (CNRS), Institut de Recherche pour le Développement (IRD), Montpellier, 34090, France; Department of Entomology, Michigan State University, East Lansing, MI 48824, United States; MIVEGEC, Université de Montpellier (UM), Centre National de la Recherche Scientifique (CNRS), Institut de Recherche pour le Développement (IRD), Montpellier, 34090, France; Department of Biological Sciences, Mississippi State University, MS 39762, United States; Department of Wildlife, Fisheries, and Aquaculture, Mississippi State University, MS 39762, United States; Fish and Wildlife Research Center, Mississippi State University, MS 39762, United States; Department of Entomology, Michigan State University, East Lansing, MI 48824, United States; Department of Wildlife, Fisheries, and Aquaculture, Mississippi State University, MS 39762, United States; Department of Wildlife, Fisheries, and Aquaculture, Mississippi State University, MS 39762, United States; Fish and Wildlife Research Center, Mississippi State University, MS 39762, United States; Department of Biological Sciences, Mississippi State University, MS 39762, United States; Department of Entomology, Michigan State University, East Lansing, MI 48824, United States; Department of Osteopathic Medical Specialties, Michigan State University, East Lansing, MI 48824, United States; Ecology, Evolution and Behavior Program, Michigan State University, East Lansing, MI 48824, United States; AgBioResearch, Michigan State University, East Lansing, MI 48824, United States; MIVEGEC, Université de Montpellier (UM), Centre National de la Recherche Scientifique (CNRS), Institut de Recherche pour le Développement (IRD), Montpellier, 34090, France

**Keywords:** infectious disease, sapronotic agent, environmental pathogen, soil, freshwater, trophic interactions, environmental disturbances, mathematical modeling, disease ecology

## Abstract

Sapronotic pathogens are constituents of complex trophic networks, such as those that structure aquatic and soil ecosystems. In such habitats, sapronotic pathogens live and reproduce among microbial consortia; they also may occasionally infect hosts and cause sapronotic disease (sapronosis). Sapronotic pathogens regroup almost all fungal microparasites and about a third of the bacterial pathogens infecting humans, including for instance nontuberculous mycobacteria. Even though sapronotic agents are naturally present in the environment, their population dynamics are unknown. Despite growing rates of sapronotic disease incidence among humans, wild, and domestic animals, very few studies have examined sapronotic transmission and dynamics in the context of spatially implicit trophic networks. Patterns of sapronotic pathogen transmission arise from complex interactions, including pathogen natural history, nonhost and host environments, and spatial and temporal scales of the system. In order to infer and ultimately predict how environmental disturbances affect trophic interactions and influence sapronotic ecology, we analysed host and nonhost species interacting as prey and as micro- and macropredators within a metacommunity context. Using a set of differential equation models, we assessed responses of environmental load dynamics of a sapronotic disease agent, i.e. a mycobacterial pathogen, within a general framework of environmental disturbance. We show that variation in top-down and horizontal interactions mediated sapronotic pathogen abundance and dynamics in the environment. Our findings indicate that habitat change and trophic interactions within these host–pathogen relationships may strongly affect sapronotic pathogen ecology through both synergistic and opposing mechanisms. This work provides for the first time an understanding of environmental disturbance consequences on trophic webs that include major sapronotic pathogens. In addition, the results provide a basis for interpreting the development of sapronotic epidemics and epizootics in the context of ecosystem modifications, particularly that of agriculture and land-use transformation. Further research of this type will provide a better understanding of the complex dynamics of sapronotic pathogens in animals and humans responding to global change.

## Graphical Abstract

**Graphical Abstract:**
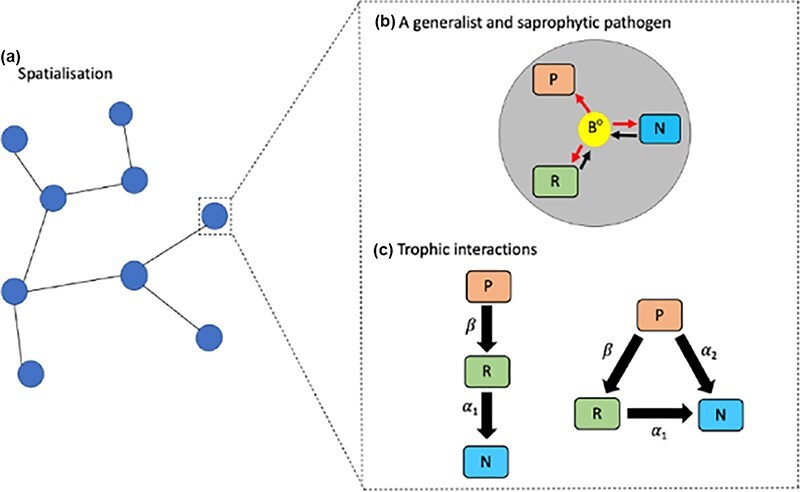
We show how environmental disturbances and food-web structure interact to shape sapronotic pathogen dynamics, offering a new framework to understand their ecology and risks under global change.

## Introduction

Human activities often cause environmental disturbances that affect biodiversity patterns across spatial scales (Dornelas et al. 2014). Characterizing how these activities may affect infectious disease risks has been challenging, notably because of the complexity of ecological interactions among biodiversity levels, ecosystem functioning and the transmission strategies that pathogens have evolved for persisting in time and space (Rohr et al. 2020). Also, pathogen transmission patterns arise from complex interactions of local transmission and the broader spatial and temporal scales of the system (Lanzas et al. 2020). Indeed, disease pathogens, by altering life-history traits of infected hosts, affect the relations between biodiversity and ecosystem functioning (Frainer et al. 2018). Reciprocally, studies have shown predator top-down control effects on parasite infection patterns (Clark et al. 2019). In analysing environmental transmission of pathogens, linking host and nonhost environment, and pathogen scales can be particularly challenging; and this can be made even more complex by taking into account human ecosystem disturbances.

Trophic interactions are important to ecosystem functioning, energy transfer, and biodiversity maintenance, and environmental disturbance can strongly impact trophic interactions in communities (Cuff et al. 2023). With environmental disturbances predicted to become increasingly more important, it is urgent to understand how changes may affect particular functional guilds of communities, and consequently influence spatial and temporal dynamics of environmental pathogen ecology (Ostfeld et al. 2008). Unfortunately, there is little disease ecology modeling work showing how environmental disturbances affecting particular trophic guilds can cascade into infectious disease transmission dynamics. This type of ecosystem-based research is crucial to broadly understanding the effects of local to global environmental changes on animal and human health (Johnson et al. 2015). Most scientific research, both empirical and theoretical, has focused on understanding the ways disease pathogens affect the existing interrelationships in food-webs, or on studying changes in food-web properties, i.e. modifying nodes and links, and their consequences in disease agents (Lafferty et al. 2008, Selakovic et al. 2014). Other work has looked at the effects of pathogens on food-webs by studying the impact on the demography of a particular trophic level (De Rossi et al. 2014). In general, the theoretical analysis of the cascading effects of environmental disturbances acting on trophic webs and impacting on the presence and population dynamics of an environmentally borne pathogen, has not been carried out (Ostfeld et al. 2008, Rossberg 2013).

Among infectious disease agents, sapronotic pathogens form an important group constituted by fungi, protozoa, and bacteria that can cause opportunistic infections in humans, wild, and domestic animals through inhalation, ingestion, and passive contacts with internal tissues via open wounds. Sapronotic pathogens can also be described as “parasitic decomposers” due to their shared ability for extracting the nutrients they need to survive and reproduce not only from alive organisms but also from decaying organic matter, which allows their environmental persistence as free-living organisms (Kuris et al. 2014, Metz et al. 2025). Expanding human habitat use and landscape change of natural ecosystems presents emerging opportunities for vertebrate sapronotic pathogens (Guégan et al. 2025a, Guerra et al. 2021), which today account for one third of the bacterial diseases affecting humans (Kuris et al. 2014). The literature on sapronotic pathogens affecting vertebrates has largely neglected their environmental living conditions to focus on infection clinical effects, which has prevented a proper understanding of the mechanisms interconnecting human-driven landscape changes to their burden increases in animal and human infections (Guégan et al. 2025b, Chevillon et al. 2024).

The climatic, abiotic, and biotic drivers of the variation in environmental abundance of sapronotic pathogens (hence in infection risks for susceptible vertebrates) remain generally poorly known, preventing accurate modeling analysis (Lanzas et al. 2020). *Mycobacterium ulcerans* (MU), the causative agent of Buruli ulcer (BU), a skin neglected tropical disease, stands as an exception since this sapronotic pathogen has drawn considerable attention by disease ecologists during the last decade (see Douine et al. 2017, Receveur et al. 2022 for reviews). Environmental disturbances, notably those of human origin (e.g. deforestation, mining, dam construction, and agricultural development), are known to be associated with increased BU disease risks in humans (Merritt et al. 2010, Guégan et al. 2020). Recent evidence indicates MU presence in aquatic habitats (e.g. mud, water column, macrophyte substrates, and carried by a wide diversity of aquatic and riverine macroorganisms) depending on seasonal periodicity (Garchitorena et al. 2015b, Morris et al. 2016a, b, Receveur et al. 2022, Chevillon et al. 2024). Since the MU DNA concentration in the water column is generally low due to the volume of water required for filtration, research has usually inferred MU presence and dynamics from patterns in MU carriage by macroorganisms (Garchitorena et al. 2015b, Receveur et al. 2022). Widely diverse taxa up to 80–90 different taxonomic orders of macroorganisms (e.g. fishes, annelids, mollusks, and aquatic and terrestrial arthropods) naturally carry MU without necessarily presenting disease symptoms (Garchitorena et al. 2015a, b, Morris et al. 2016b, Receveur et al. 2022). Time-series analyses assessing seasonal variations in the distribution and carriage of MU among aquatic and riverine MU carriers in Cameroon, Central Africa showed that direct acquisition of MU from contaminated water bodies was the main mode of transmission to humans compared to alternative routes (Garchitorena et al. 2015a). This is also likely the case for most MU carriers, which offer microhabitats of variable quality for further MU replication in/on their bodies, even if trophic transmission cannot firmly been ruled out (Garchitorena et al. 2015a, b, Receveur et al. 2022). MU is also frequently found in mud close to aquatic habitats (Williamson et al. 2012), with the soil at the interface between freshwater and riparian ecosystems thought to represent the ecological niche for this bacillus (Guégan et al. 2025b, Falkinham 2009). The rare field studies that had included direct MU quantification in nearby (distance <500 m) soil and water samples (Wiliamson et al. 2012, Bratschi et al. 2014, Dogbe et al. 2025) brought strong support to this hypothesis. These studies consistently reported maximal estimates in bacterial loads that were one-to-two magnitude orders higher in soils than in nearby water bodies. For a given sampling date and a given locality, these studies also reported patchy MU distributions in soils but narrow variation range in MU abundance among nearby water samples. Overall, these patterns suggest a water-borne diffusion of MU bacilli of telluric origin that homogenizes MU loads among nearby water bodies connected by water flow. Rainfall during the rainy season in intertropical regions, soil leaching and flooding all contribute to MU release into watersheds (Guégan et al. 2025b), which explains the seasonal nature of both the abundance of MU in aquatic systems and human BU cases (Garchitorena et al. 2015a, Vargas Campos et al. 2025). It is noteworthy that such a seasonal dynamics also implies that the MU carriage by macroorganisms and MU demographical dynamics as a free-living bacterium are not enough to sustain long-term persistence of MU in the water bodies constituting infectious risks for humans.

In natural aquatic ecosystems where sapronotic pathogens circulate, these functional trophic groups correspond to well-defined ecological entities. Micropredators typically include small predatory invertebrates, such as aquatic insects (e.g. odonates and hemipterans) or small crustaceans, which frequently prey on primary consumers and are often reported among the most common carriers of sapronotic bacteria. Macropredators, in contrast, correspond to larger predators such as fish, amphibians, or large predatory arthropods that feed on both prey and smaller predators. Introducing these examples provides biological context for the functional groups incorporated in our model and reflects the trophic diversity observed in real sapronotic systems. More generally, sapronotic pathogens are thought to persist not only through reservoir hosts but also through environmental reservoirs, whose precise nature remains poorly resolved in the literature (Kuris et al. 2014). These environmental compartments may episodically supply aquatic systems during seasonal flooding or heavy rainfall events, thereby contributing to the maintenance and persistence of bacterial populations across years.

We previously developed a spatially explicit metacommunity model to investigate, at the scales of a whole river catchment and for multiple years, how the seasonal variations in rainfall, hence in river flow, and in the abundance of biotic communities developing along riversides, may durably amplify MU abundance downstream (Sylla et al. 2024). The present study uses a similar mathematical framework to investigate how ecological disturbances destabilizing food-webs involving both putative MU carriers and noncarriers may affect MU abundance at finer scales, i.e. a set of interconnected flooded alluvial areas in-between two rainy seasons. For the sake of simplicity, we call these flooded areas in rainy seasons “ponds” or “patches” hereafter. We first explore how the structure of trophic interactions influences the persistence and amplification of the aquatic abundance of the sapronotic pathogen at such fine spatio-temporal scales. We then analyse the cascading effects of local and regional environmental disturbances affecting higher trophic levels onto the abundance of the MU free-living stage. Rather than aiming to predict a specific empirical system, this study proposes a conceptual and mechanistic modeling framework to understand how spatial structure, trophic complexity, and ecological disturbances interact to shape generalist pathogen dynamics. While MU is our main example because its ecology is better understood, the model’s structure and equations are kept general. This framework is readily adaptable to other sapronotic pathogens, provided that the diversity of potential carrier organisms is known. In such cases, only specific parameters (e.g. growth, attachment, and death rates) must be modified, while the fundamental structure of the model remains unchanged. By illustrating the types of mechanisms that can drive persistence or collapse of a generalist sapronotic pathogen in spatially structured ecosystems, we offer a theoretical foundation that can inform future extensions incorporating empirical data. This perspective highlights the need for greater attention to the environmental life of sapronotic disease agents affecting vertebrates, and supports the development of mathematical modeling tools dedicated to this underexplored but ecologically important category of human and animal pathogens.

## Materials and methods

### Model formulation

**Figure 1:**
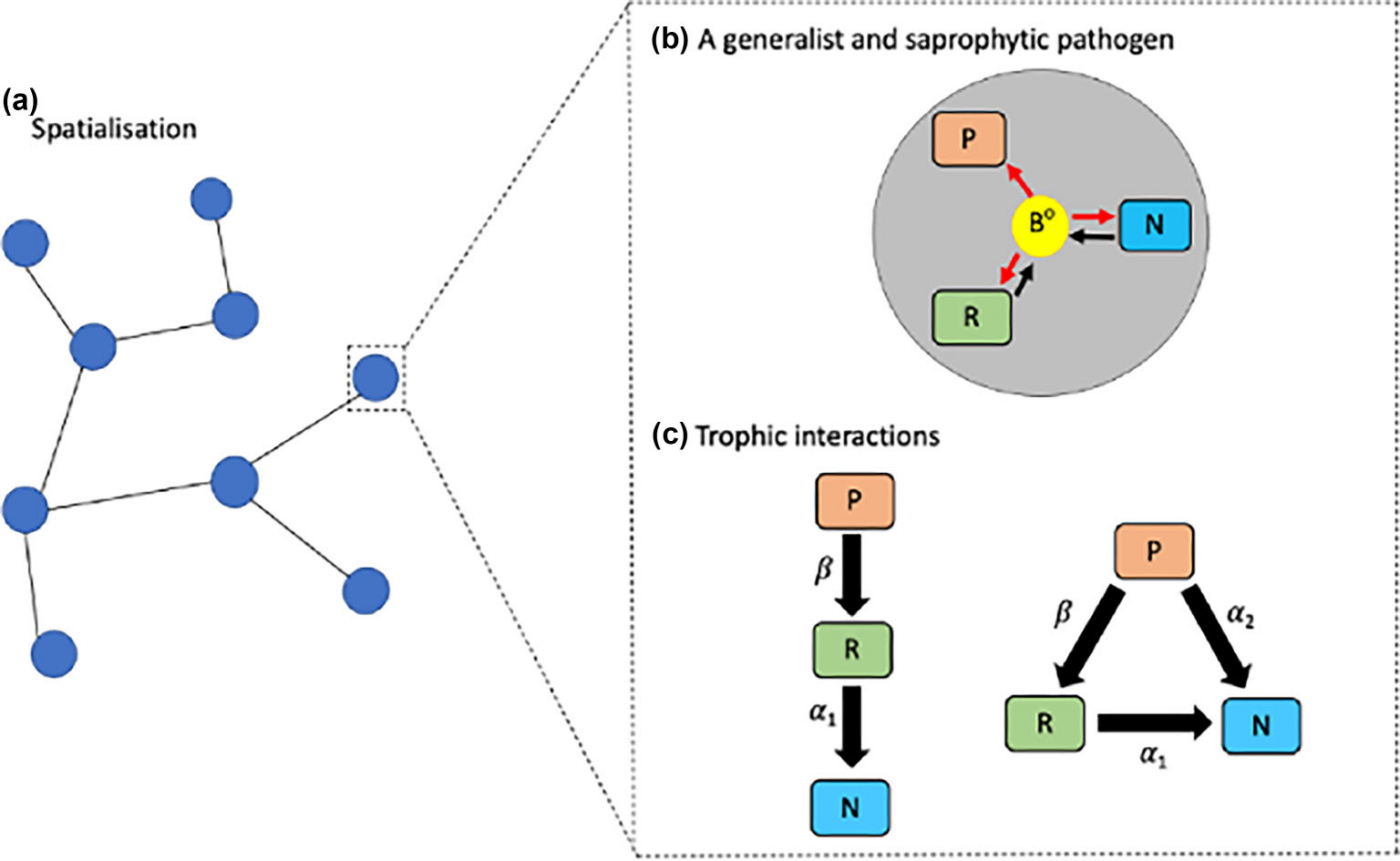
Schematic representation. State variables and parameters are defined in Table 1. Panel (a) illustrates the metacommunity in space: it includes nine aquatic habitat patches (represented by blue dots) that differ in productivity levels and are interconnected (connections represented by black lines), which allows macropredator migration across patches. Panels (b) and (c) describe the interspecific interactions occurring within each local patch as illustrated on panel a. Panel (b) focuses on the characteristics of a bacterial pathogen that is both generalist and saprophytic, hence occurring within patches as both free-living bacteria (B^0^) and in association with different organisms (i.e. P, N, and R for macropredators, micropredators, and prey, respectively). The red and black arrows respectively represent the attachment rates of free-living bacteria to living biomass and the releases of free-living bacteria from dead infected organisms when their bodies are decomposing. Panel (c) shows scenarios of trophic interactions considered depending on the diet of the macropredators when micropredators are only feeding on prey. Macropredators can be specialists feeding only on micropredators (on the left) or generalists feeding on micropredators and prey (on the right). We defined *α_1_* and *α_2_* as parameters describing the attack rates onto prey by micro- and macropredators, respectively, and by *β* the attack rate of macropredators onto micropredators. This allowed switching from the trophic chain scenario, illustrated on the left, to the intraguild predation scenario, figured on the right, simply by manipulating the value taken by *α_2_* (null on the left and nonnull on the right).

We propose a spatially implicit metacommunity model, where a generalist and sapronotic bacterial pathogen coexists and interacts with three functional groups of free-living species: (1) prey, representing excellent resources for pathogen survival and reproduction, and two groups of predators, hereafter referred to as (2) micro- and (3) macropredators. Figure 1 illustrates the interspecific interactions considered. Table 1 details the functions and parameters of the metacommunity model. Although micropredators exclusively feed on prey, we consider two scenarii depending on macropredator diet. The first is a simple trophic chain, with macropredators feeding on micropredators that, in turn, consume prey. The second, called intraguild predation (Arim and Marquet 2004), stipulates that micro- and macropredators may share a common resource: under that scenario, macropredators are generalist feeding on both preys and micropredators with diet shifting when micropredators become locally rare. Trophic interactions allow pathogen transmission from infected species to their predators. We incorporated the fact that the bacterium is a sapronotic pathogen by considering its population dynamics as a free-living bacillus and explicitely formulating the local exchanges between its free-living stage and stages, where it is associated to alive and dead organisms (i.e. either prey or micropredator since macropredator represents a dead-end for the sapronotic pathogen in present study). Free-living bacterial cells may directly attach to living organism surfaces (called biomass hereafter), and then can be released from infected organisms upon death (hereafter refer to as bacterial saprophytic transmission). Bacterial doubling times and carrying capacity of biomass-associated bacteria differed between the three functional groups (Table 1). We modeled a metacommunity model with nine different local aquatic patches, i.e. ponds, differing in productivity level, i.e. in the nutriments available for prey survival and growth. We integrated this heterogeneity in the model by conserving among-patches differences in prey growth rate (*r_i_*) and carrying capacity (*K_i_*). The present model is developed as a conceptual tool to explore how the structure of trophic interactions and disturbance patterns affect the dynamics of such a sapronotic pathogen in both time and space.

**Table 1:** Functions and parameters of the metacommunity model. The third column indicates the values taken by default for the different parameters across experiments. When no value is illustrated, it means that parameter is either a vector or a function described in text.

Notations	Descriptions	Default values
	State functions	
*N_i_*	Density of prey species in patch *i*	
*R_i_*	Density of micropredator species in patch *i*	
*P_i_*	Density of macropredator species in patch *i*	
*B_i_^N^*	Density of prey-associated bacteria in patch *i*	
*B_i_^R^*	Density of micropredator-associated bacteria in patch *i*	
*B_i_^P^*	Density of macropredator-associated bacteria in patch *i*	
*B_i_^o^*	Density of free-living bacteria in patch *i*	
	Parameters	
*r_i_*	Intrinsic growth rate of prey species in patch *i*	–
*K_i_*	Environmental carrying capacity of preys in patch *i*	1
μ	Natural mortality of preys	0.05
α_1_	Maximal attack rate of micropredators onto preys	0.4
α_2_	Maximal attack rate of macropredators onto preys	0 or 0.25
β	Maximal attack rate of macropredators onto micropredators	0.25 or 0.5
e_1_,e_2_,e_3_	Constants of the Beddington–DeAngelis functional response	0.1
b	Constant of the Beddington–DeAngelis functional response	1
h	Constant of the Beddington–DeAngelis functional response	1
c_1_	Food conversion efficiency of preys into micropredator births	0.7
c_2_	Food conversion efficiency of preys into macropredator births	0.7
c_3_	Food conversion efficiency of micropredators into macropredator births	0.7
d_1_	Natural death rate of micropredators	0.05
d_2_	Natural death rate of macropredators	0.05
m_i_(t)	Migration rate of macropredator in patch *i*	–
q*_ji_*	Probability that an individual from patch *i* migrates to patch *j*	1/8
ξ	Bacterial attachment rate on prey biomass	0.4
γ	Bacterial attachment rate on micropredator biomass	0.13
ρ	Bacterial attachment rate on macropredator biomass	0.06
s_N_	Half-saturation constant of prey-associated substrate	1
s_1_	Half-saturation constant of micropredator-associated substrate	1
s_2_	Half-saturation constant of macropredator-associated substrate	1
θ	Growth rate of prey-associated bacteria	0.05
λ	Growth rate of micropredator-associated bacteria	0.02
η	Natural mortality of prey-associated bacteria	0.03
ν_1_	Natural mortality rate of micropredator-associated bacteria	0.2
ν_2_	Natural mortality rate of macropredator-associated bacteria	0.3
Λ(t)	Time-dependence free-living bacteria growth rate	–
δ	Natural mortality rate of free-living bacteria	0.03
*κ*	Bacilli release rate from decomposing bodies of infected preys	3
*σ*	Bacilli release rate from decomposing bodies of infected micropredators	2

In patch *i*, we denoted by functions of time t: *N_i_* the density of prey, *R_i_* the density of micropredator species, *P_i_* the density of macropredator species, *B_i_^N^* the density of prey-associated bacteria, *B_i_^R^* the density of micropredator-associated bacteria, *B_i_^P^* the density of macropredator-associated bacteria, and by *B_i_^o^* the density of free-living bacteria in the aquatic environment. We assume that the prey population obeys a classical logistic growth, with *r_i_* referring to the intrinsic growth rate and *K_i_* to the environmental carrying capacity. Due to predation, the prey population size decreases by a quantity proportional to prey and predator populations. Here, the predator–prey interactions are modeled via Beddington–DeAngelis’ functional response (Beddington 1975, DeAngelis et al. 1975). This functional response is an enhancement of the well-known Holling’s type II functional response including a term representing interference among predators. Increasing the density of consumers (i.e. predators) also reduces the consumption rate of the resource (i.e. preys). Several studies in the ecological literature have also considered this type of response (Cantrell et al. 2004, Tripathi et al. 2015, Ghanbari and Kumar 2019, Ji and Wang 2022).

Using the functions and parameters described in Table 1, we formulated the model as follows. The dynamics of biomass for the three functional groups in patch *i* are given by:


(1) \begin{eqnarray*}
\frac{{d{N_i}}}{{dt}} = {r_i}{N_i}\left( {1 - \frac{{{N_i}}}{{{K_i}}}} \right) - \mu {N_i} - \frac{{\left( {{\alpha _1}{R_i} + {\alpha _2}{P_i}} \right){N_i}}}{{1 + {e_1}{R_i} + {e_2}{P_i} + b{N_i}}}.
\end{eqnarray*}

(2) \begin{eqnarray*}
\frac{{d{R_i}}}{{dt}} = \frac{{{c_1}{\alpha _1}{R_i}{N_i}}}{{1 + {e_1}{R_i} + {e_2}{P_i} + b{N_i}}} - {d_1}{R_i} - \frac{{\beta {P_i}{R_i}}}{{1 + {e_3}{P_i} + h{R_i}}}.
\end{eqnarray*}

(3) \begin{eqnarray*}
\frac{{d{P_i}}}{{dt}} &=& \frac{{{c_2}{\alpha _2}{P_i}{N_i}}}{{1 + {e_1}{R_i} + {e_2}{P_i} + b{N_i}}} - {d_2}{P_i} + \frac{{{c_3}\beta {P_i}{R_i}}}{{1 + {e_3}{P_i} + h{R_i}}}\\
&&- {m_i}{P_i} + \sum\limits_{j \ne i} {{m_j}{q_{ij}}{P_j}}.
\end{eqnarray*}

Parameters *α_1_* and* α_2_* denote, respectively, the attack rate of micro- and macropredators onto preys. Parameter β represents the predation rate of macropredators onto micropredators. Predator growth clearly depends on food consumption. We thus define *c_1_, c_2_*, and *c_3_* as the respective food conversion efficiency of predators, representing thus the number of newborn predators resulting from their consumption. In absence of prey, micro- and macropredator densities decay exponentially at rate *d_1_* and *d_2_*, respectively. The term μ*N_i_* describes the prey loss due to natural mortality. Our model also incorporates macropredator migration events across the different local communities. At each time *t*, a fraction of macropredators *m_i_*(*t*) leaves patch *i* to other surrounding patches. A part *q_ji_* of these emigrants choose patch *j* as destination. Thus, the flux of biomass from patch *i* to patch *j* at time *t* is *m_i_(t)q_ji_P_i_(t)*. Summing the immigrants coming from all the other patches to patch *i* provides the last term figuring in equation (3).

The dynamics of prey-associated, micropredator-associated, and macropredator-associated bacteria in patch *i* are respectively given by:


(4) \begin{eqnarray*}
\frac{{dB_i^N}}{{dt}} &=& \frac{{\xi {N_i}B_i^o}}{{{s_N} + {N_i}}} + \frac{{\theta {N_i}B_i^N}}{{{s_N} + {N_i}}} - \left( {\eta + \mu } \right)B_i^N\\
&&- \frac{{\left( {{\alpha _1}{R_i} + {\alpha _2}{P_i}} \right)B_i^N}}{{1 + {e_1}{R_i} + {e_2}{P_i} + bB_i^N}}.
\end{eqnarray*}

(5) \begin{eqnarray*}
\frac{{dB_i^R}}{{dt}} &=& \frac{{{\gamma _i}{R_i}B_i^o}}{{{s_1} + {R_i}}} + \frac{{\lambda {R_i}B_i^R}}{{{s_1} + {R_i}}} - \left( {{\nu _1} + {d_1}} \right)B_i^R\\
&& - \frac{{{\alpha _1}{R_i}B_i^N}}{{1 + {e_1}{R_i} + {e_2}{P_i} + bB_i^N}} - \frac{{\beta {P_i}B_i^R}}{{1 + {e_3}{P_i} + hB_i^R}}.
\end{eqnarray*}

(6) \begin{eqnarray*}
\frac{{dB_i^P}}{{dt}} &=& \frac{{\rho {P_i}B_i^o}}{{{s_2} + {P_i}}} - \left( {{\nu _2} + {d_2}} \right)B_i^P + \frac{{{\alpha _2}{P_i}B_i^N}}{{1 + {e_1}{R_i} + {e_2}{P_i} + bB_i^N}}\\
&&+ \frac{{\beta {P_i}B_i^R}}{{1 + {e_3}{P_i} + hB_i^R}} - {m_i}B_i^P + \sum\limits_{j \ne i} {{m_j}{q_{ij}}B_j^P}.
\end{eqnarray*}

The intrinsic growth rate of host-associated bacteria depends upon the density of predators and prey biomasses as substrates for saprophytic bacterial development. The parameters η, ν_1_, and ν_2_ correspond, respectively, to the natural mortality rate of prey-associated, micropredator-associated, and macropredator-associated bacteria. Here, we used Michaelis–Menten’ type functional response to describe bacilli attachment to biomass, which is proportional to both population biomass and free-living bacterial densities. We defined the maximum success rates for bacilli attachment on prey, micropredators, and macropredators as *ξ*, γ_i_, and *ρ*, respectively. The biomass-associated bacteria are able to replicate on alive biomass until surface saturation concentration is reached. Focusing for instance on prey-associated bacteria, the Michaelis–Menten’ type functional response is thus given by *θN_i_/(N_i_+*s_N_*)*, where *θ* is the maximum production rate of prey-associated bacteria, and s_N_ is the half-saturation constant in this type of microhabitat. The same functional response was used for predator-associated bacterial reproduction. Saprophytic bacilli growing on the surface of living biomass are released when the bodies of dead infected organisms are decomposing, which thus produce free-living bacteria (Godfray et al. 1999, Kunttu et al. 2009, Merikanto et al. 2018). These phenomena, assumed to occur only in prey- and micropredator guilds (i.e. macropredators are considered dead-ends for the pathogen), are here described by parameters *κ* and *σ. *Note that, aside such releases, biomass mortality reduces the concentration of biomass-associated bacteria that are respectively described by terms –μ*B_i_^N^, -d_1_B_i_^R^*, and *-d_2_B_i_^P^* in equations (4), (5), and (6), respectively. Similarly, while feeding on the prey present in patch *i*, predators locally decrease the number of prey-associated bacteria. In our model, we further incorporated the changes in the local densities of biomass-associated bacteria resulting from migration of macropredators among connected patches (see above).

The dynamics of free-living bacteria in any patch *i* is given by:


(7) \begin{eqnarray*}
\frac{{dB_i^o}}{{dt}} &=& \left( {\Lambda - \delta } \right)B_i^o - \frac{{\xi {N_i}B_i^o}}{{{s_N} + {N_i}}} - \frac{{{\gamma _i}{R_i}B_i^o}}{{{s_1} + {R_i}}}\\
&&- \frac{{\rho {P_i}B_i^o}}{{{s_2} + {P_i}}} + \kappa \mu B_i^N + \sigma {d_1}B_i^R,
\end{eqnarray*}

where Λ(t) and δ are the reproduction and natural mortality rates of free-living bacteria, respectively. The body decomposition of infected organisms, either preys or micropredators, release saprophytic bacteria in the environment, increasing thus the local density in free-living bacteria in the patch when these organisms died (see terms *κ*μ*B_i_^N^*and *σd_1_B_i_^R^* in equation (7).

The metacommunity dynamics result from macropredators dispersal among-patches. We considered this dispersal to be active, depending on the local availability of resources. In the trophic chain scenario (Fig. 1c, left), we have described the migration rate, *m_i_*(*t*), as a function inversely proportional to the local density of micropredators:


(8) \begin{eqnarray*}
{m_i}\left( t \right) = \frac{u}{{1 + {R_i}\left( t \right)}},
\end{eqnarray*}

where *u* is the maximal dispersal rate. In the alternative scenario considering macropredators as generalists (Fig. 1c, right), we have described the migration rate, *m_i_*(t), as a function inversely proportional to both local densities of prey and micropredators:


(9) \begin{eqnarray*}
{m_i}\left( t \right) = \frac{u}{{1 + {R_i}\left( t \right) + {N_i}\left( t \right)}}.
\end{eqnarray*}

These definitions ensure that the migration rate of macropredators will increase when local food abundance decreases. For simplicity, we suppose that the pool of emigrants leaving the patch *i* has equal probability to reach any other local habitats (q*_ji_* = 1/(*n*−1)).

Regarding environmental disturbances, we focused on cases where they promote sudden increases in predator mortality, hence instantaneous decreases in predator densities (*P_i_* and/or *R_i_*). We also considered perturbations that, in addition to their sudden and direct impact on predator mortality, reduce the growth rate in preys, *r_i_*, relatively to the value determined by the primary productivity level of the affected patch (see below, the third simulating experiment). Given differences among simulation experiments considered in the present paper, the modeling methodology is further developed within each section referring to a particular experiment.

### Simulation parameters

Model parameters were not empirically calibrated, but chosen to reflect ecologically plausible values and ensure diverse but interpretable dynamical behaviors at fine spatial and temporal scales. We simulated a metacommunity composed of nine interconnected local sites. We set the resource vector describing prey growth rate among sites as *r* = [0.15, 0.17, 0.2, 0.23, 0.25, 0.28, 0.31, 0.35, 0.38], allowing spatial heterogeneity in resource dynamics. We simulated free-living bacteria reproduction as simple Weibull’s distribution: Λ(*t*) = 4$ \times $Weibull(*t*,100,4). This time-dependent formulation generates a transient growth pulse that peaks and then progressively decreases, causing the intrinsic reproduction rate of free-living bacteria to become negligible as environmental conditions deteriorate during the dry period. Table 1 presents the values taken by default across all simulations by other parameters. Parameter values were chosen to ensure biologically plausible dynamics, but no empirical calibration was attempted. This parameterization is consistent with the model’s conceptual purpose, which is not to reproduce a particular empirical system, but rather to identify the key mechanisms by which spatial and trophic structure can mediate sapronotic pathogen dynamics. Simulations are conducted over a 1-year temporal window encompassing a rainy season followed by a dry season, in order to investigate short-term pathogen persistence. All simulations and numerical integrations were conducted in MATLAB ver. 8.6, © 1994–2025 The MathWorks, Inc.

### Simulation scenarios overview

The present study does not focus on the long-term equilibria applying on the ecological metacommunities of free-living species that may carry the sapronotic pathogen but on the transient impacts of ecological disturbances affecting these communities of potential carriers onto the abundance in the free-living environmental stage of the pathogen (i.e. the stage determining human infectious risks) across nearby local ponds. We designed four distinct simulation experiments to explore how ecological disturbances affect the dynamics of this sapronotic pathogen embedded within a trophically structured metacommunity of potential carriers. These scenarios vary in the spatial scale of disturbance (local versus regional), its temporal nature (instantaneous versus prolonged), the trophic groups affected (prey, micropredators, and macropredators), and the structure of trophic interactions (specialist versus generalist macropredators). Table 2 summarizes the main features and objectives of each simulation. The combination of multiple trophic configurations and spatial disturbance regimes enabled us to probe a wide range of ecological conditions and disturbance types. Although inspired by MU ecology, the framework is deliberately abstracted and applicable to a broader class of other sapronotic systems (see an important list in Kuris et al. 2014). Unless otherwise specified, all simulation outputs and figures presented in this study depict the temporal dynamics in free-living bacterial density (expressed in free-living cells per water volume), which represents the environmental stage of the sapronotic pathogen and constitutes the focus of our analysis.

**Table 2:** Summary of simulation scenarios used in this study.

Experiment	Disturbance type	Affected trophic levels	Spatial scale	Macropredator diet	Main objective
1. Local disturbance	Instantaneous reduction in predator density	Either macropredators, or both micro- and macropredators	Local (single patch)	Specialist or generalist	Assess the differences in local bacterial load responses to sudden reduction in predator density between food-web structures
2. Regional disturbance reducing predators densities (only macropredators disperse)	Synchronous or asynchronous events with either similar or different impact across patches	Micro- and macropredators	Regional (all patches)	Specialist or generalist	Explore the effects of timing, severity, and spatial heterogeneity of disturbances that suddenly reduce local predator densities
3. Regional disturbance reducing predators densities and prey growth (only macropredators disperse)	Synchronous events, with variable impacts on predator densities, and temporary reducing prey growth	All functional groups	Regional (all patches)	Specialist or generalist	Examine the impact of the duration during which the basal resource (i.e. prey) is affected
4. Relaxed dispersal constraints	Same as previously, but dispersal abilities extended to micropredator and prey	Variable	Metacommunity	Specialist or generalist	Evaluate robustness of model outcomes to broader dispersal assumptions

### Comparative disturbance metrics

To better synthesize and compare the effects of different disturbance regimes on environmental pathogen dynamics, we defined four summary metrics extracted from the time-series of free-living bacterial density in each simulation, which allowed us to compare how disturbances affected the density in free-living bacteria across aquatic sites relatively to the bacterial density expected in the same community in absence of disturbance. These metrics are as follows.

- The maximum transient deviation (Δ_peak) represents the maximum absolute difference in free-living bacterial density between the disturbed and undisturbed scenarios. This metric quantifies the peak intensity of the bacterial response to the disturbance.
- T_peak is the date when the deviation in bacterial response from the expectations in absence of disturbance is maximal.
- The resilience time (T_resilience) is the number of days required for the system to return to within 5% of its predisturbance dynamic equilibrium. It is important to note that the apparent return to equilibrium observed in our simulations does not represent a permanent steady state, but rather the stabilization of the bacterial load following a transient perturbation. This metric captures the speed at which the system recovers from disturbance.
- The postdisturbance equilibrium level (B_eq_post) is the average density in free-living bacteria when it recovers a new steady state after the disturbance. This metric reflects the long-term impact of the disturbance on bacterial abundance.

These metrics were calculated for representative simulations across different regimes to facilitate comparison of the system behavior under varying disturbance intensities and spatial scales.

## Results

Numerical simulations are proposed with the aim of understanding how disturbing local communities affects, via their direct and indirect impacts on species communities, the environmental contamination by a bacterial pathogen that is both generalist and saprophytic (i.e. the density in free-living bacterial cells representing human infection risks). For each model scenario, we randomly chose the timing of disturbances, and repeated simulations with different starting dates of disturbances and variable severity impacts on local communities. We explored the properties of the model by investigating how such variation in timing, spatial scale, and/or severity of disturbances affect free-living bacteria density. In other terms, the present study examines the sensibility of ecological disturbances onto the environmental abundance of the pathogen computed for the same model structure (i.e. a given food-web structure interconnecting potential carriers and a given set of interactions between the pathogen and potential carriers. For each model scenario, we ran the simulations long enough to encompass the two phases that follow disturbance occurrence, i.e. a first phase along which the food-web remains destabilized, and a second one when the abundance of the free-living stage of the pathogen in water is stabilized. In the two first experiments, local or regional environmental disturbances are instantaneous events that only affect predatory guilds: predator densities in disturbed patch(es) are recovering just after the environmental disturbance as a function of the local resources. In the third experiment, environmental disturbances reduce the densities of predator but also affects prey growth, with this latter effect lasting for several days or weeks, promoting delays in both prey and predator recoveries relatively to expectations for undisturbed habitats. In these former experiments, the only functional guild dispersing across patches is that of macropredator, representing a dead-end for the pathogen. The fourth experiment allowed dispersal of the micropredator and prey, which provides pathogen resources as both living and dead organisms. In disturbance regimes involving predator reductions, individuals were removed instantaneously from the system, and no carcass-derived biomass was retained. Hereafter, for the sake of brevity, we have selected the most interesting and informative findings.

### Experiment 1: local ecological disturbance (i.e. affecting a single patch)

**Figure 2:**
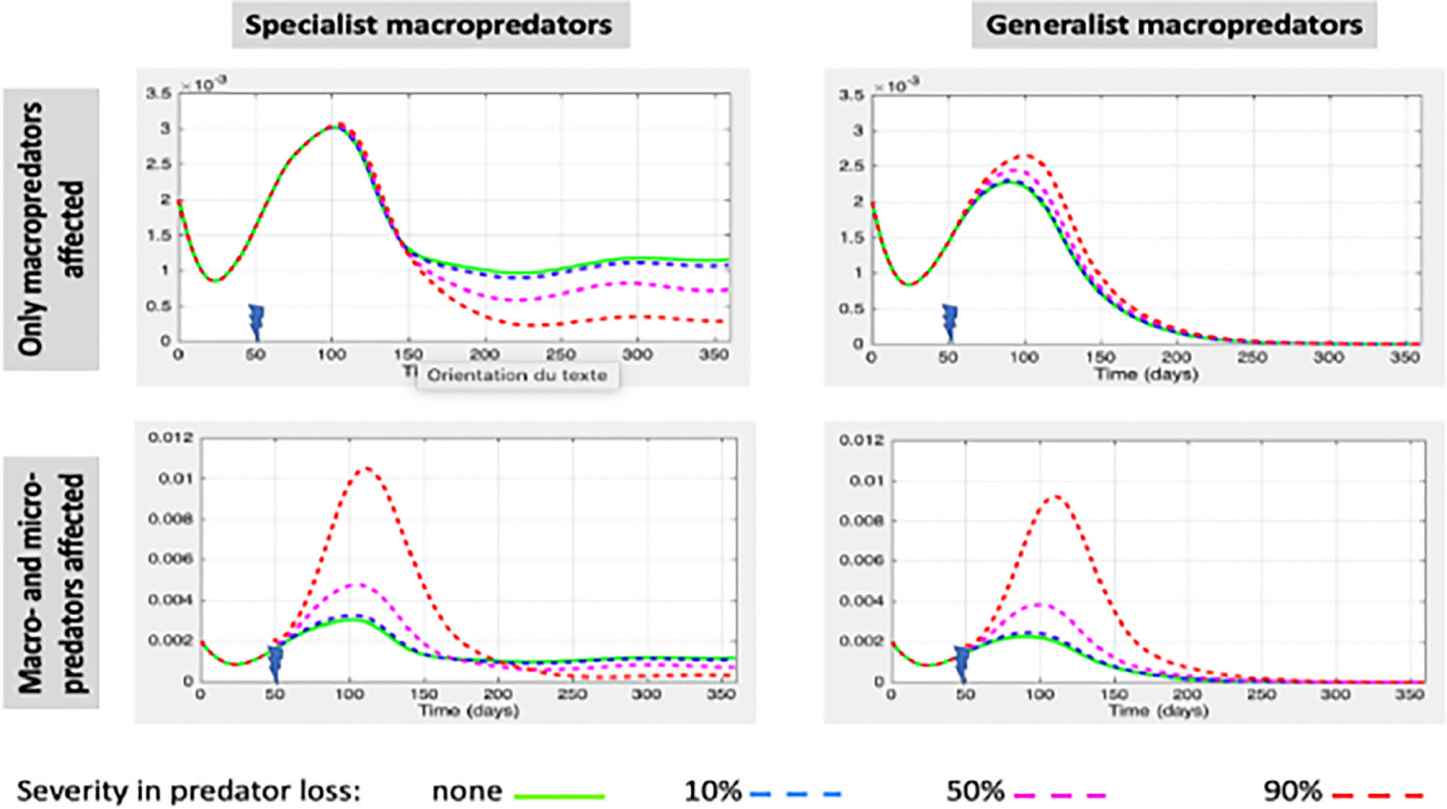
Effects of local disturbances onto the local dynamics in free-living bacteria density. *x*-axes represent time and *y*-axes free-living bacteria density (i.e. number of cells per volume of water). In all panels, green curves represent the local density in free-living bacteria in absence of perturbation. Other curves refer to the dynamics in free-living bacteria for an instantaneous reduction in predator densities at *t* = 50, when disturbance occurs, ranging from 10% to 90%. The perturbations affect only macropredators in top panels, and both micro- and macropredators in bottom panels. Macropredators are specialists in left panels and generalists in right ones. Predation parameters used in trophic chain scenarios are *α_2_*= 0 and β = 0.5; they are *α_2_* = 0.25 and β = 0.25 otherwise.

Figure 2 illustrates cases of local environmental disturbance that occur when the fluctuating density in saprophytic bacteria was naturally rising. For a given community at *t* = 0, four possibilities of disturbance exist. The environmental disturbance may affect only macropredators (Fig. 2, top panels) or both macro- and micropredators (Fig. 2, bottom panels). Macropredators may be specialist (left panels) or generalist (right panels). In each panel, the green curve represents the temporal dynamics in free-living bacteria density that one would have observed in absence of disturbance. Green curves are thus identical in the two right panels (left panels, respectively) but obviously different between left and right panels. Other lines represent the dynamics in the density of free-living bacteria observed after disturbance.

In all panels, the most obvious impact of disturbance onto the dynamics in free-living bacteria density relies to the period along which the local food-web remains destabilized. There is a temporary increase in sapronotic bacteria along that period, which results from a temporary excess of the inputs from the decomposing bodies of previously infected organisms relatively to the observations in absence of disturbance. For a given food-web structure (i.e. for panels within the same column in Fig. 2), such an excess is much higher when the disturbance affects both predatory guilds, enriching thus the community in unconsumed preys (bottom panels) relatively to the case where disturbance only affect macropredators (top panels). For a given impact of disturbance (i.e. for panels within the same raw), the food-web structure matters on the long-term response to local disturbance. Indeed, in trophic chain scenarii (i.e. involving only specialist predators), the postdisturbance equilibrium translates in a reduction in free-living bacteria density relatively to expectations in absence of any disturbance of the local community. This is not the case for food-web involving generalist macropredators: there, the pre- and postdisturbance equilibria of the system result in similar dynamics in free-living bacteria density.

### Experiment 2: regional ecological disturbance affecting predators

Here, we consider the case of regional ecological disturbance so that (i) every patch is the subject of local disturbance once a year and (ii) within-patch disturbances evenly affect micro- and macropredators. Patches still differ from one another in primary productivity (hence in prey logistic growth). Local disturbances can occur as synchronous events across all patches or as asynchronous events that include time lags between disturbances affecting distinct local patches. In addition, disturbances may differ in the severity of predator reductions they induced. We thus simulated synchronous and asynchronous events with homogeneous (i.e. all local communities suffer the same reduction in both micro- and macropredator densities, set at 10%, 50%, and 90%) and heterogeneous effects across patches (reduction in predator densities’ range: 0%–100%).

**Figure 3:**
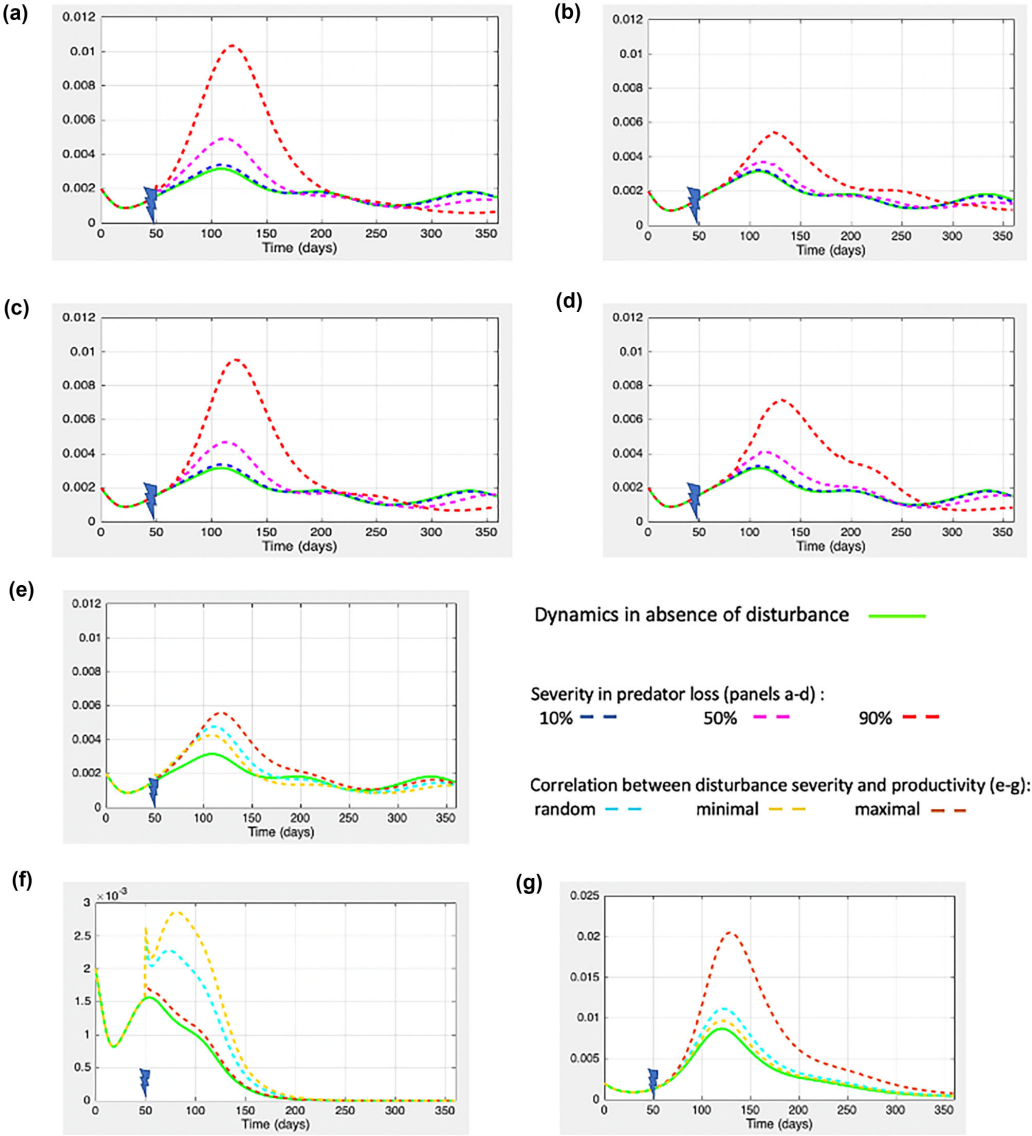
Impact of a succession of within-patches disturbances (trophic chain scenario). The diet of specialist macropredators is described by *α_2_* = 0 and β = 0.5. First local disturbance occurs at *t* = 50 days. Panels (a–e) consider the regional scale of the entire metapopulation. Green curves refer to the regional dynamics in free-living density observed in absence of disturbances and others to those observed after successions of within-patches disturbances. In (a–d) panels, all local disturbances have homogeneous impact on within-patches predator densities. These local events are synchronized across patches in panel a. In panel (b), the vector describing random timing of local disturbances across patches is *t* = [50, 123, 180, 346, 50, 211, 81, 271, 92, 183]. Time lags between successive disturbances last 5 and 20 days in panels c and d, respectively. In panel (e), local disturbances are synchronized events with heterogeneous impacts on predator densities across patches described by the vector [0.3371,0.1622,0.7943,0.3112,0.5285,0.1656,0.6020,0.2630,0.6541]. Panels (f) and (g) focus on the within-patches responses observed in the least and most productive patches, respectively.

Figure 3 shows the simulation results in free-living bacterial load obtained when a disturbance affects specialist macropredators. Figure 3(a–d) provide evidence of the importance of time lags between successive disturbances affecting distinct patches with homogeneous severity. Specifically, synchronous events (panel a) or short delays (panel c) between successive disturbances events lead to larger temporary increases in regional average in free-living bacteria density. For instance, following a 50% reduction in predator densities, Δ_peaks corresponds to ~152% of the bacterial density expected at *t* = T_peak in undisturbed communities (panels a–c), but does not exceed 140% in panels b–d.

Next, we assessed the importance of heterogeneity in predator losses resulting from synchronous regional disturbance on free-living bacteria density. Here, there are two sources of heterogeneity among patches: differences in primary productivity *r* (ranging from 0.15 to 0.38) and differences in predator losses (randomly selected from 0% to 100%). In order to examine the interplay between the two sources of heterogeneity, we explored the dynamics in the regional average in free-living bacteria density when the correlation between them was random, minimal, or maximal (Fig. 3, panels e and f). At local scale, the impact of the heterogeneity in disturbance severity exceeds that of the heterogeneity in productivity (see the yellow curve in f panel and/or the red curve in g panel). Unsurprisingly, at the regional scale, the highest rebound in free-living bacteria density occurs when the correlation between both sources of among-patches heterogeneity is maximal (Fig. 3e).

Results obtained for generalist macropredators are similar and presented in supplementary materials (Fig. S1). It is nevertheless noteworthy that, as previously observed at local scale (Fig. 2), the diet of the macropredators affects the long-term response of the metacommunity to a succession of local disturbances. In one instance, when both micro- and macropredator guilds are specialist, the main difference between pre- and postdisturbances equilibria at metacommunity scale translates into a reduction in free-living bacteria density (Fig. 3). In the second instance, when macropredator are generalist, the long-term response of the metacommunity to a succession of local disturbances tend to be the same as that observed in absence of disturbance (Fig. S1).

### Experiment 3: regional ecological disturbance also affecting prey

The results presented so far concern instantaneous disturbance effects that only affect predator guilds. However, some disturbances, such as pollution resulting from gold mining, may affect each of the species present in aquatic communities. Here, we explore cases where disturbances affect all functional groups although differently. Synchronous disturbances reduce predator densities, but promote a temporary or lasting reduction in prey population growth.

**Figure 4:**
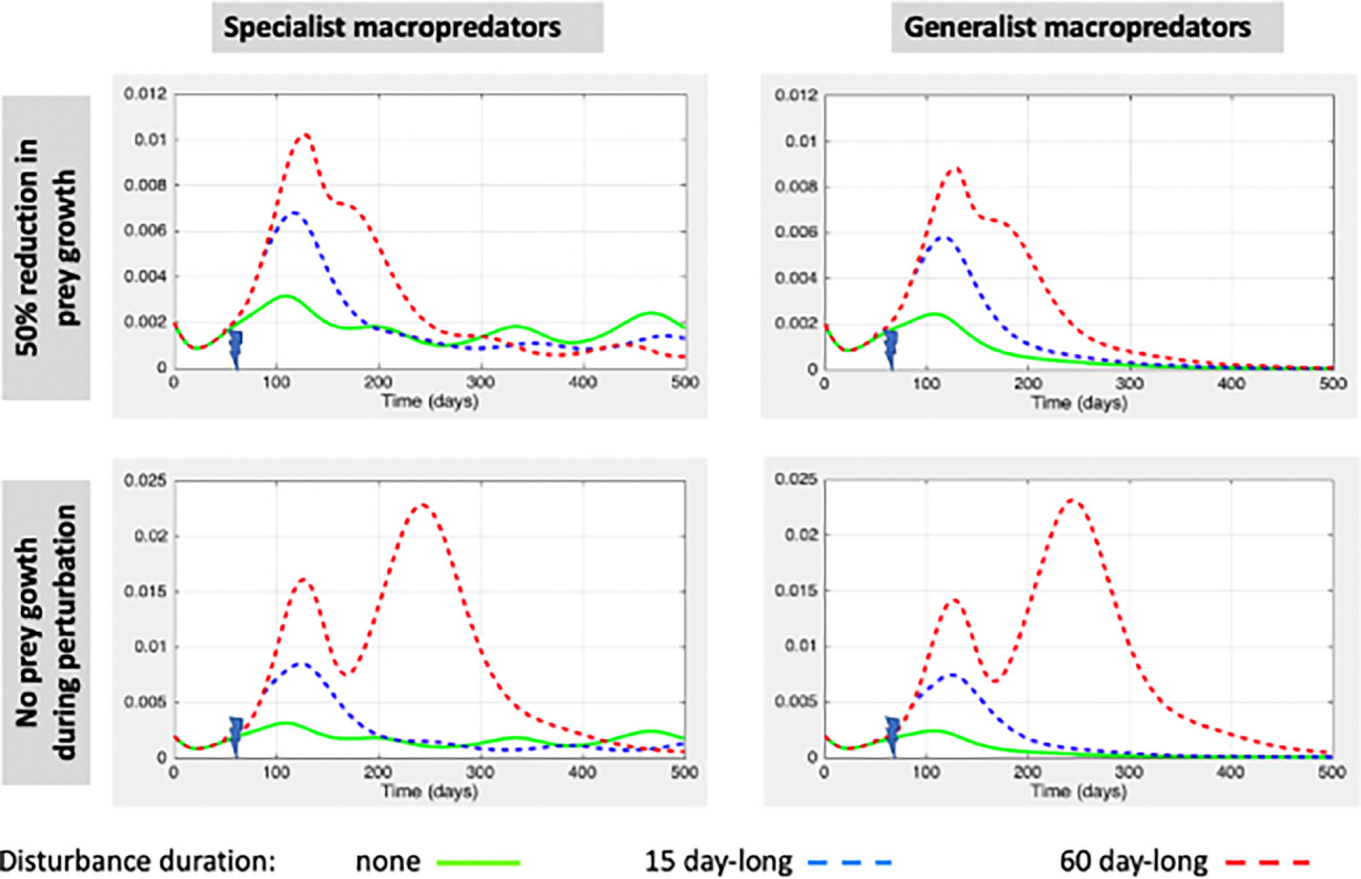
Regional disturbance effects on prey, micro- and macropredators that impact density of free-living bacteria over the metacommunity. *x*-axes represent time; disturbances occur at *t* = 69 days. *y*-axes refer to the regional averages in free-living bacteria density. Macropredators are specialist (*α_2_* = 0 and β = 0.5) in left panels and generalist (*α _2_* = 0.25 and β = 0.25) in right panels. Green curves refer to the regional average dynamics in saprophyte density observed in absence of disturbances. Those are identical in both left panels (right panels, respectively) but differ between right and left panels. Disturbances promote instantaneous reductions in predator. The vector describing the heterogeneity across patches in predator losses is [0.6663, 0.5391, 0.6981, 0.6665, 0.1781, 0.1280, 0.9991, 0.1711, 0.0326]. The blue and red curves refer to the regional responses in free-living bacteria density to disturbances blocking prey growth for 15 and 60 days, respectively. The reduction in prey growth during disturbances is 50% and 100% in top and bottom panels, respectively. Please note that the scale of the *y*-axes in bottom panels exceeds those referring to regional average in bacterial loads in Figs 2 and 3.

We carried out a sensitivity analysis on the duration of the blockage period of prey growth after disturbance for different impacts of local reproduction rate *r_i_*. Figure 4 shows the results of simulations in cases where *r_i_* is reduced either by 50% (top panels) or by 100% (bottom panels), and where macropredators are either specialist (left panels) or generalist (right panels). We found that the regional average in free-living bacterial density depends on how long and to which extent disturbances affects prey growth. It is noteworthy that the extent in the reduction in prey growth (here, 50% versus 100%) have larger impacts on metacommunity responses relatively to the period duration of such effects on prey (here, 15 days versus 60 days).

### Experiment 4: relaxing dispersal constraints on functional groups including species competent for the pathogen survival and growth

The first three experiments focused on cases where macropredators, which are dead-end hosts for the bacterial pathogen, constitute the only functional ecological group that include species dispersing across the metacommunity. We tested the dependency of the results obtained under this hypothesis by recomputing simulations for each experiment while assuming that species from the three trophic levels disperse across the metacommunity (everything else remaining unchanged). We assumed passive and nonoriented dispersal for preys, so that a fixed percentage of prey emigrate from each local site and evenly distribute among other local sites. For micropredators, we assumed active migration that depends on the local resources in preys (i.e. obeying to the same rules than those defined for specialist macropredators; see equation 8 above).

Figures S3–S6 detail results for experiments 1–3 under two dispersal hypotheses for prey and micropredator (i.e. cases A and B). In case A, we fixed prey dispersal at 1% and maximal dispersal rates of micro- and macropredator at 5% and 70%, respectively. In case B, we fixed prey dispersal at 6% and maximal dispersal rates of micro- and macropredator at 10% and 70% for micro- and macropredators, respectively. Comparing those to Figs 2 and 3 and Fig. S1 evidence that the signals observed when only macropredator disperse remain if species from all functional groups disperse. Introducing dispersal of prey and micropredator nonetheless tend to make perturbations promoting lower and delayed peaks in the abundance of free-living bacteria relatively to cases where only macropredator disperse.

### Comparative assessment of disturbance effects

We used a set of resilience metrics to summarize and compare the system responses to disturbance across different experiments (Table 3). The peak amplification due to the ecological disturbance (Δ_peak) was highest under increase-gradient and synchronized disturbances, reflecting stronger trophic release. Asynchronous and gradient-decreasing disturbances led to faster return to equilibrium (lower T_resilience), while high-frequency uniform scenarios showed delayed stabilization. These results reinforce the role of disturbance synchrony and heterogeneity in shaping both the intensity and duration of the amplification in the abundance in free-living cells of the sapronotic pathogen.

**Table 3:** Synthetic resilience metrics for some disturbance scenarios. This table summarizes key dynamic indicators for a subset of disturbance scenarios drawn from Experiments 2 and 3. In all cases, the first disturbance occurred at *t* = 50 days. Disturbances in Experiment 2 reduced local predator densities by 50%, while in Experiment 3 they combined predator reductions with a temporary (15-day) decrease in prey growth rate by 50%. The percentages given in parentheses for Δ_peak expressed the deviation in disturbed metacommunity relatively to the expectations at *t* = T_peak in absence of any disturbance (everything else being equal).

	Comparative metrics in bacterial responses to ecological disturbances			
Scénarios ()	Δ_peak (cells/water volume)	T_peak (days)	T_resilience (days)	B_eq_post (cells/water volume)
Experiment 2—specialist macropredators				
Synchronous events	0.0018 (+56%)	115	140	0.0011
Asynchronous events	0.0006 (+20%)	123	175	0.0012
High-frequency uniform disturbances with a constant interval of 5 days between patch successive disturbances	0.0016 (+52%)	118	178	0.0012
Low-frequency uniform disturbances with a constant interval of 20 days between patch successive disturbances	0.0011 (+40%)	130	176	0.0013
Random disturbances distribution in time	0.0016 (+51%)	113	155	0.0012
Synchronous events with minimal correlation between heterogeneity in productivity and the severity in disturbance effects	0.0011 (+34%)	107	115	0.0011
Synchronous events with maximal correlation between patch heterogeneity in productivity and the severity in disturbance effects	0.0026 (+89%)	124	179	0.0014
Experiment 2—generalist macropredators				
Synchronous events	0.0016 (+64%)	114	279	0.0004
Asynchronous events	0.0006 (+240%)	123	289	0.0004
High-frequency uniform disturbances with a constant interval of 5 days between patch successive disturbances	0.0014 (+60%)	117	279	0.0004
Low-frequency uniform disturbances with a constant interval of 20 days between patch successive disturbances	0.0010 (+51%)	130	278	0.0004
Random disturbances distribution in time	0.0015 (+59%)	112	273	0.0004
Synchronous events with minimal correlation between heterogeneity in productivity and disturbance effects	0.0010 (+40%)	99	266	0.0004
Synchronous events with maximal correlation between patch heterogeneity in productivity and disturbance effects	0.0024 (+109%)	125	298	0.0003
From experiments 3				
Specialist macropredator—duration 15-days	0.0035 (+119%)	124	139	0.0010
Generalist macropredator—duration 15-days	0.0033 (+152%)	126	283	0.0003

## Discussion

Empirical studies show that predators can influence pathogen emergence and transmission, even while reducing prey and vector abundance (Cohen et al. 1990). Yet, despite growing recognition that multitrophic interactions affect pathogen disease ecology, few empirical or theoretical studies have examined how trophic complexity and spatial structure jointly influence environmental pathogen dynamics (see Clark et al. 2019). This gap limits our ability to understand and predict how disturbances propagate through food-webs and affect pathogen persistence and dispersion. In this study, we developed a mechanistic and conceptual model to explore how local and regional disturbances of a three-level trophic network affect the abundance of a sapronotic and generalist pathogen. The present framework is entirely adaptable to sapronotic or environmental pathogens whose distribution depends on their interactions with organisms distributed across several trophic levels. In the present study, the quality of the microhabitats offered by potential carriers to the sapronotic pathogen decreased from prey to micropredator, and again from micro- to macropredator. This directly derived from the knowledge acquired on MU. While reviewing the literature on MU ecology, Receveur et al (2022) listed, with indication of their feeding guilds, the organisms identified as MU carriers across unbiased field surveys [see Table S2 of Receveur et al. (2022), with “community” as sampling method]. This revealed a rarity of top-predators among MU carriers, which are very often arthropods belonging to several lower trophic levels. Our hypothesis is also in line with the literature on the generalist strains involved in human gastroenteritis (e.g. *Enterobacter, Escherichia coli, Shigella*, and *Salmonella* strains), which recently supported change in the understood the main driver of human exposure from the carriage in livestock (Key et al. 2020) to the carriage by cultivated plants (Teplitski and de Moares 2018, Quagliariello and Marvasi 2024). As recently discussed (Guégan et al. 2025b, Chevillon et al. 2024), this hypothesis likely extends to other sapronotic and generalist pathogens; although further research is needed on the full range of biotic interactions to determine whether a greater dependence on organisms belonging to lower trophic levels can be considered a general rule.

Our results identify three core mechanisms that govern sapronotic pathogen responses to disturbance: (i) trophic release following predator loss, (ii) spatial synchronization or desynchronization of disturbance events, and (iii) asymmetric recovery rates among trophic levels. Together, these mechanisms interact to determine whether the sapronotic pathogen undergoes transient amplification, persistent suppression, or resilience in both time and space.

First, we show that local disturbances that reduce predator densities can destabilize trophic control, creating a transient window during which these bacterial pathogens may be amplified. This transitory effect is amplified when both predator guilds are affected, and when macropredators are specialists. In these cases, prey escapes predation more effectively, increasing decomposition inputs and resource availability for sapronotic bacteria. However, relative to generalist ones, the transitory period is shorter with specialist macropredators and the postdisturbance dynamic equilibrium resulting in a reduced production of free-living bacteria than expected in an absence of disturbance. Generalist predators, in contrast, confer functional redundancy that buffers the disturbance effects, so that the system slowly returns back to its predisturbance dynamic equilibrium. These findings support the idea that trophic flexibility and redundancy are critical for ecological resilience and disease ecology (McCann 2000, Elmqvist et al. 2003).

Second, at the metacommunity scale, regional disturbances that are synchronized, rather than asynchronous, in time produce larger amplification peaks for bacterial pathogens. This is due to additive trophic release across local patches, whereas asynchrony allows unaffected patches to compensate for others in recovery. Moreover, the spatial heterogeneity of disturbance severity interacts with patch productivity, such that disturbances that disproportionately affect highly productive patches (i.e. where prey growth rate is maximal), lead to more pronounced regional amplification. These results underscore the importance of both the spatial structure and regional synchrony of local disturbance events in shaping sapronotic pathogen dynamics.

Third, we find that delayed recovery of prey populations—such as might occur under chemical pollution or climatic stress—can extend the destabilization phase and further amplify pathogen abundance in the environment. The magnitude of prey growth suppression had a greater effect on pathogen load than the duration of the delay, suggesting nonlinear threshold effects in trophic recovery. These findings illustrate how disturbance impacts that reach basal trophic levels can have cascading effects on pathogen persistence and development.

Fourth, dispersal of prey and micropredators across the metacommunity acts as a spatial buffer, smoothing local fluctuations and promoting faster recovery. Systems where only macropredators disperse show stronger and longer-lasting local amplification, particularly in specialist food webs. Thus, species mobility and trophic structure jointly mediate the system’s ability to absorb and recover from disturbance. The comparative metrics confirm that spatial synchrony and trophic structure jointly influence both the magnitude and persistence of sapronotic pathogen amplification. In particular, synchronous disturbances regimes led to higher peaks and delayed recovery, whereas asynchronous or spatially heterogeneous disturbances promoted faster stabilization. Then, these results support the idea that resilience is strongly shaped by trophic flexibility and spatial buffering.

Our model is intentionally theoretical, designed to reveal general mechanisms rather than predict outcomes for a specific disease agent system. Nonetheless, it aligns perfectly well with strong empirical insights from studies on *M. ulcerans* and the skin neglected disease it causes in humans (Receveur et al. 2022, Chevillon et al. 2024). It also matches other observations on distinct disease systems, such as vector-borne ones, including hantavirus and Lyme disease (Tian and Stenseth 2019, Levi et al. 2012, Clark et al. 2019). By integrating food-web theory with spatial and disturbance ecology, our framework offers a novel insight for assessing the vulnerability of sapronotic and other environmental pathogen systems to ecological disruption. It notably shows that environmental disturbances and changes may strongly affect such pathogen dynamics (see Table 4) having the counter-intuitive effect of amplifying the presence and abundance of such pathogens in the environment, and therefore having significant implications for public and planetary health. This last finding is particularly consistent with observations and estimates made concerning an increase in *M. ulcerans* loads in environments disturbed by human activities (Guégan et al. 2025b).

**Table 4:** Summary of findings for disease ecology.

Experiment	Main results for ecosystems	Main results for the free-living density of the sapronotic pathogens
1. Local disturbance	Destabilization of local trophic web	Increase in bacterial load locally except when predators are generalists
2. Regional disturbance	Destabilization of regional trophic webs, depending on synchronization or not of disturbance events	Higher peaks when regional disturbances are synchronized
3. Regional + prey impact	Destabilization of regional trophic webs, impacting prey growth	Longer transitory periods with high bacterial density
4. Relaxed dispersal	Acting as spatial buffering with faster recovery	Longer but lower bacterial peaks depending on synchronicity and spatial heterogeneity of disturbances events

Future work could expand this model by incorporating new empirical data, stochastic events, climate trends, or specific host species dynamics. Extending simulations over longer temporal scales would also allow for the examination of chronic disturbance regimes, feedbacks, tipping points, and regime shifts more realistically. This would be particularly valuable for understanding the emergence and persistence of such sapronotic pathogens under global change scenarios (see Walsh et al. 2019, Guerra et al. 2021).

Although our study is theoretical, the predictions can be empirically evaluated using controlled ecological experiments. Mesocosm or microcosm systems could be designed to manipulate the intensity and timing of environmental disturbances, predator densities, or prey productivity while monitoring the abundance of free-living and biomass-associated sapronotic or other environmental bacteria through molecular quantification (e.g. qPCR or metagenomic sequencing). Such approaches would make it possible to verify the transient amplification of such pathogens following predator loss, the buffering role of biomass-associated pathogens, and the effects of spatial synchrony/asynchrony among disturbances. Integrating experimental manipulations with our modeling framework would therefore provide a powerful way to bridge theory and data, and to assess how environmental pressures shape the ecology of such sapronotic and environmental disease agents.

In summary, our findings support the growing view that multitrophic and spatially structured ecological networks are central to understanding disease ecology. Disturbance regimes, food-web architecture, and functional recovery all interact to shape pathogen outcomes within metacommunities. Our conceptual model thus offers a new foundation for future empirical or predictive studies linking biodiversity change, environmental perturbation, human activities and infectious disease risk for both humans and animals.

## Conclusion

Our results highlight that sapronotic pathogens are highly dependent on the ecological context of their reservoir communities and can be strongly regulated by prey–predator interactions within trophic networks. Our model shows that transient changes in free-living bacterial abundance are particularly sensitive to the magnitude and spatial synchrony of environmental disturbances. These findings underline the importance of considering trophic and spatial structures when assessing the ecological dynamics of these sapronotic and environmental pathogens. More generally, this work contributes a theoretical framework for studying how environmental pressures influence pathogen persistence and dynamics in aquatic or soil habitats. Future developments should integrate empirical data and experimental validation to refine model predictions and better anticipate the ecological conditions that favor disease emergence and outbreaks in animals and humans.

## Data availability

Script and codes are available online (https://zenodo.org/records/15839730; Sylla et al. 2024a); supplementary figures are available online (https://zenodo.org/records/11083413; Sylla et al. 2024b). No new data were analysed in support of this research.

## Supplementary Material

fiag006_Supplemental_File
