# Supplementary material for: Environmental disturbances of trophic interactions and their impacts on a multihost sapronotic pathogen
Source: FEMS Microbiol Ecol. 2026 Jan 28;102(2):fiag006. doi: 10.1093/femsec/fiag006 (PMC12883986; doi:10.1093/femsec/fiag006)
Supplement: fiag006_Supplemental_File [file fiag006_supplemental_file.pptx]

## Slide 1
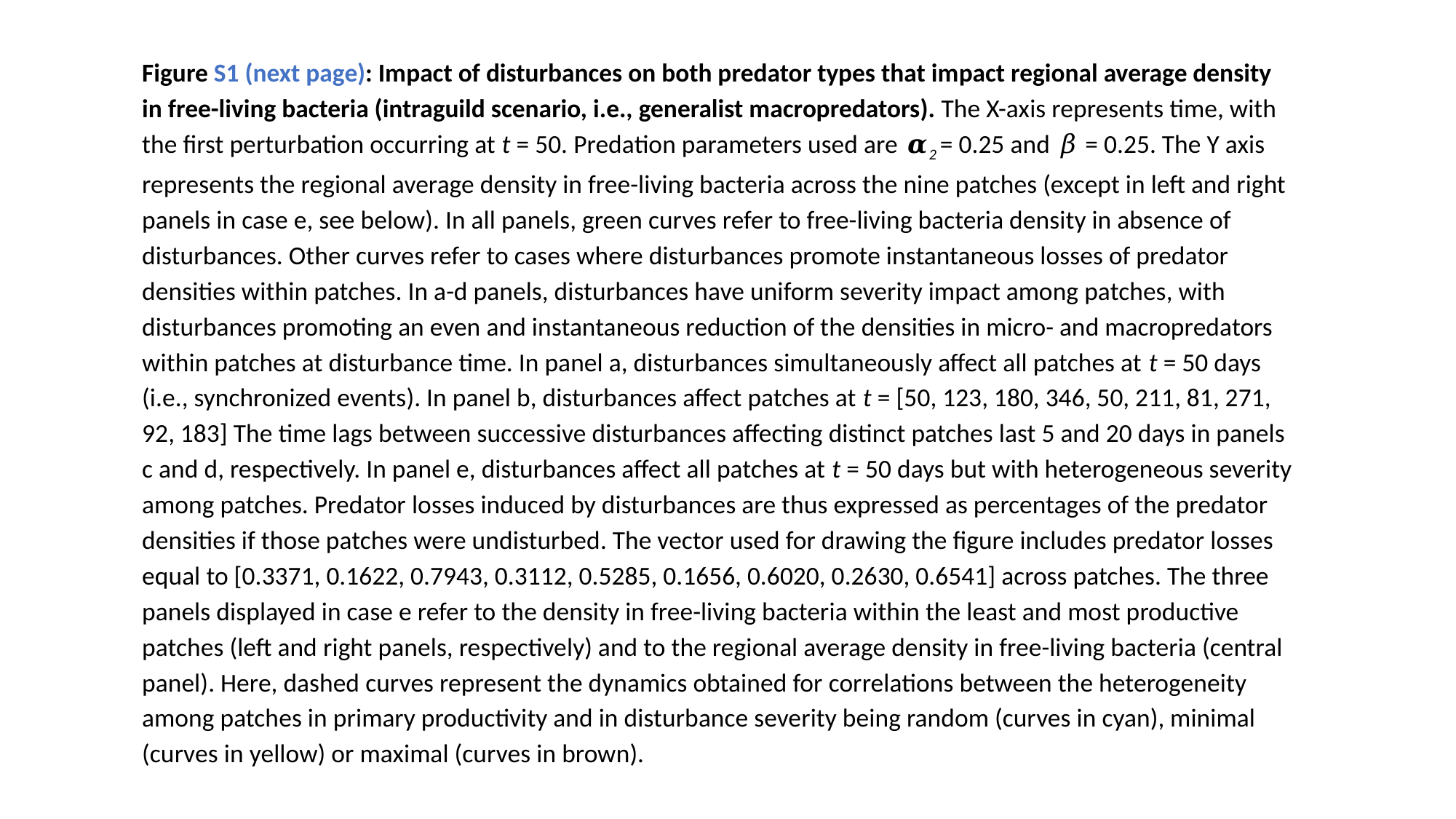

Figure S1 (next page): Impact of disturbances on both predator types that impact regional average density in free-living bacteria (intraguild scenario, i.e., generalist macropredators). The X-axis represents time, with the first perturbation occurring at t = 50. Predation parameters used are 𝜶2 = 0.25 and 𝛽 = 0.25. The Y axis represents the regional average density in free-living bacteria across the nine patches (except in left and right panels in case e, see below). In all panels, green curves refer to free-living bacteria density in absence of disturbances. Other curves refer to cases where disturbances promote instantaneous losses of predator densities within patches. In a-d panels, disturbances have uniform severity impact among patches, with disturbances promoting an even and instantaneous reduction of the densities in micro- and macropredators within patches at disturbance time. In panel a, disturbances simultaneously affect all patches at t = 50 days (i.e., synchronized events). In panel b, disturbances affect patches at t = [50, 123, 180, 346, 50, 211, 81, 271, 92, 183] The time lags between successive disturbances affecting distinct patches last 5 and 20 days in panels c and d, respectively. In panel e, disturbances affect all patches at t = 50 days but with heterogeneous severity among patches. Predator losses induced by disturbances are thus expressed as percentages of the predator densities if those patches were undisturbed. The vector used for drawing the figure includes predator losses equal to [0.3371, 0.1622, 0.7943, 0.3112, 0.5285, 0.1656, 0.6020, 0.2630, 0.6541] across patches. The three panels displayed in case e refer to the density in free-living bacteria within the least and most productive patches (left and right panels, respectively) and to the regional average density in free-living bacteria (central panel). Here, dashed curves represent the dynamics obtained for correlations between the heterogeneity among patches in primary productivity and in disturbance severity being random (curves in cyan), minimal (curves in yellow) or maximal (curves in brown).

## Slide 2
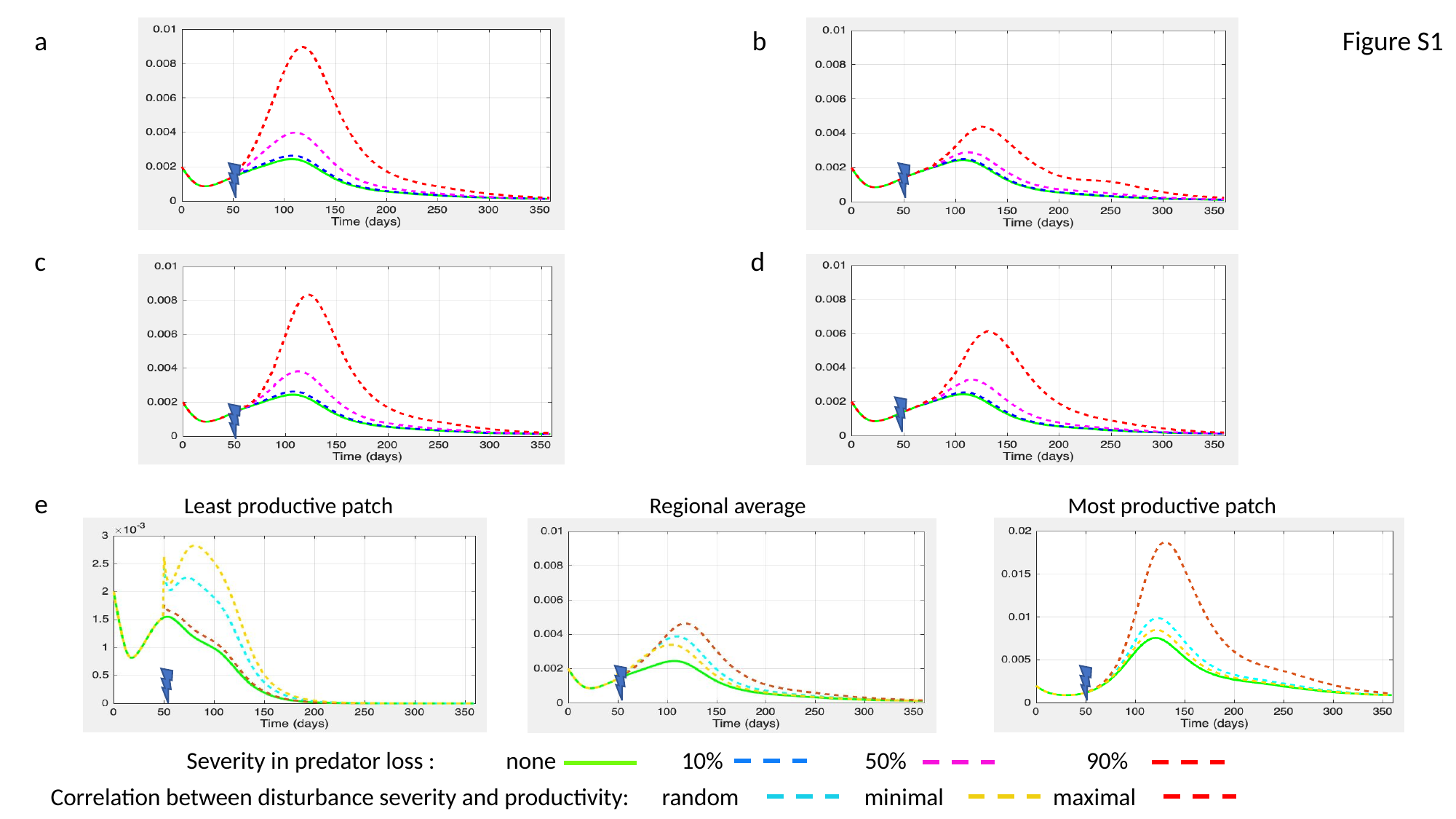

a
b
Figure S1
c
d
e
Least productive patch
Regional average
Most productive patch
Severity in predator loss : none 10% 50% 90%
Correlation between disturbance severity and productivity: random minimal maximal

## Slide 3
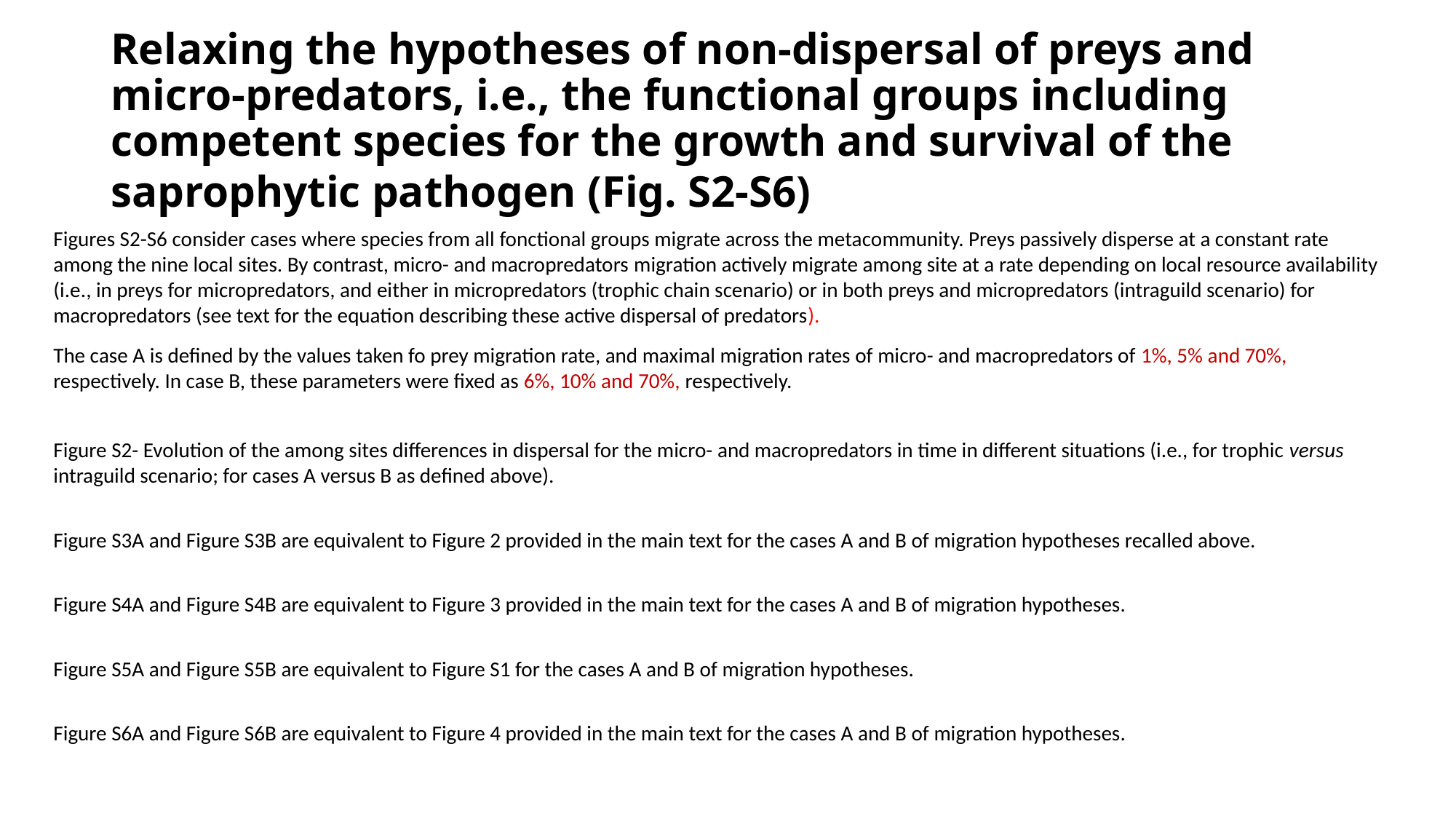

# Relaxing the hypotheses of non-dispersal of preys and micro-predators, i.e., the functional groups including competent species for the growth and survival of the saprophytic pathogen (Fig. S2-S6)
Figures S2-S6 consider cases where species from all fonctional groups migrate across the metacommunity. Preys passively disperse at a constant rate among the nine local sites. By contrast, micro- and macropredators migration actively migrate among site at a rate depending on local resource availability (i.e., in preys for micropredators, and either in micropredators (trophic chain scenario) or in both preys and micropredators (intraguild scenario) for macropredators (see text for the equation describing these active dispersal of predators).
The case A is defined by the values taken fo prey migration rate, and maximal migration rates of micro- and macropredators of 1%, 5% and 70%, respectively. In case B, these parameters were fixed as 6%, 10% and 70%, respectively.
Figure S2- Evolution of the among sites differences in dispersal for the micro- and macropredators in time in different situations (i.e., for trophic versus intraguild scenario; for cases A versus B as defined above).
Figure S3A and Figure S3B are equivalent to Figure 2 provided in the main text for the cases A and B of migration hypotheses recalled above.
Figure S4A and Figure S4B are equivalent to Figure 3 provided in the main text for the cases A and B of migration hypotheses.
Figure S5A and Figure S5B are equivalent to Figure S1 for the cases A and B of migration hypotheses.
Figure S6A and Figure S6B are equivalent to Figure 4 provided in the main text for the cases A and B of migration hypotheses.

## Slide 4
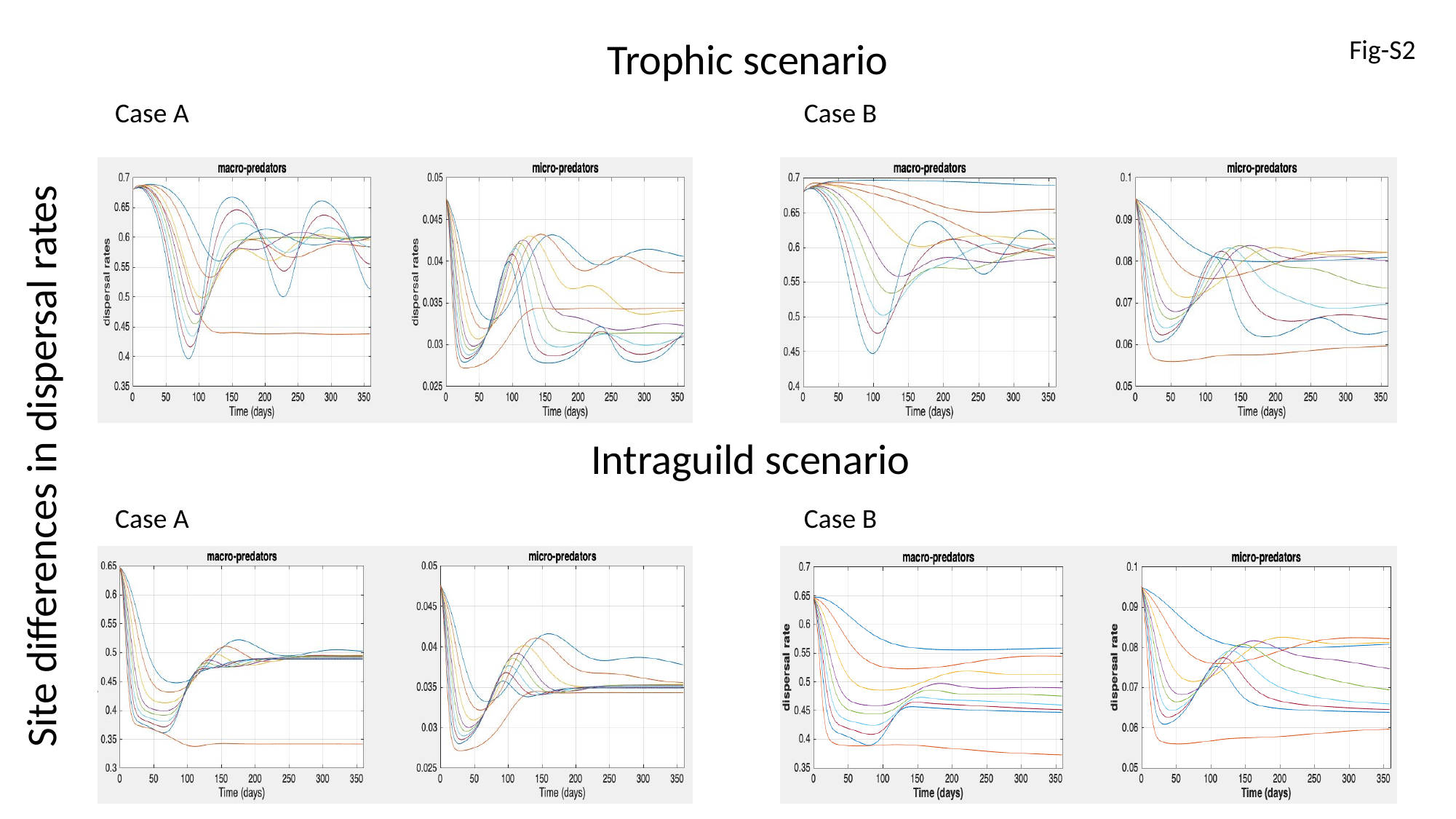

Trophic scenario
Fig-S2
Case A
Case B
Site differences in dispersal rates
Intraguild scenario
Case A
Case B

## Slide 5
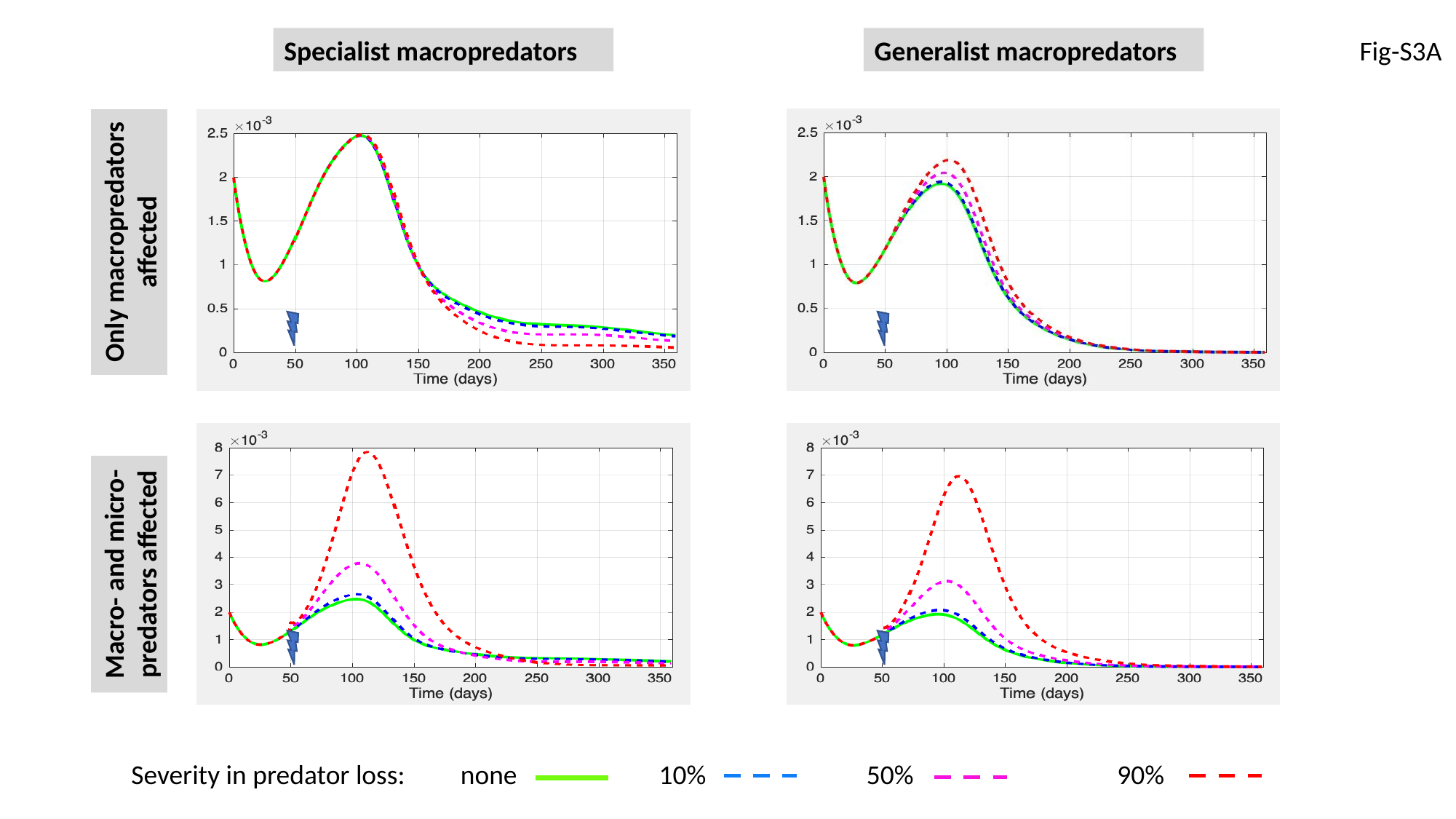

Specialist macropredators
Generalist macropredators
Fig-S3A
Only macropredators affected
Macro- and micro-
predators affected
Severity in predator loss: none 10% 50% 90%

## Slide 6
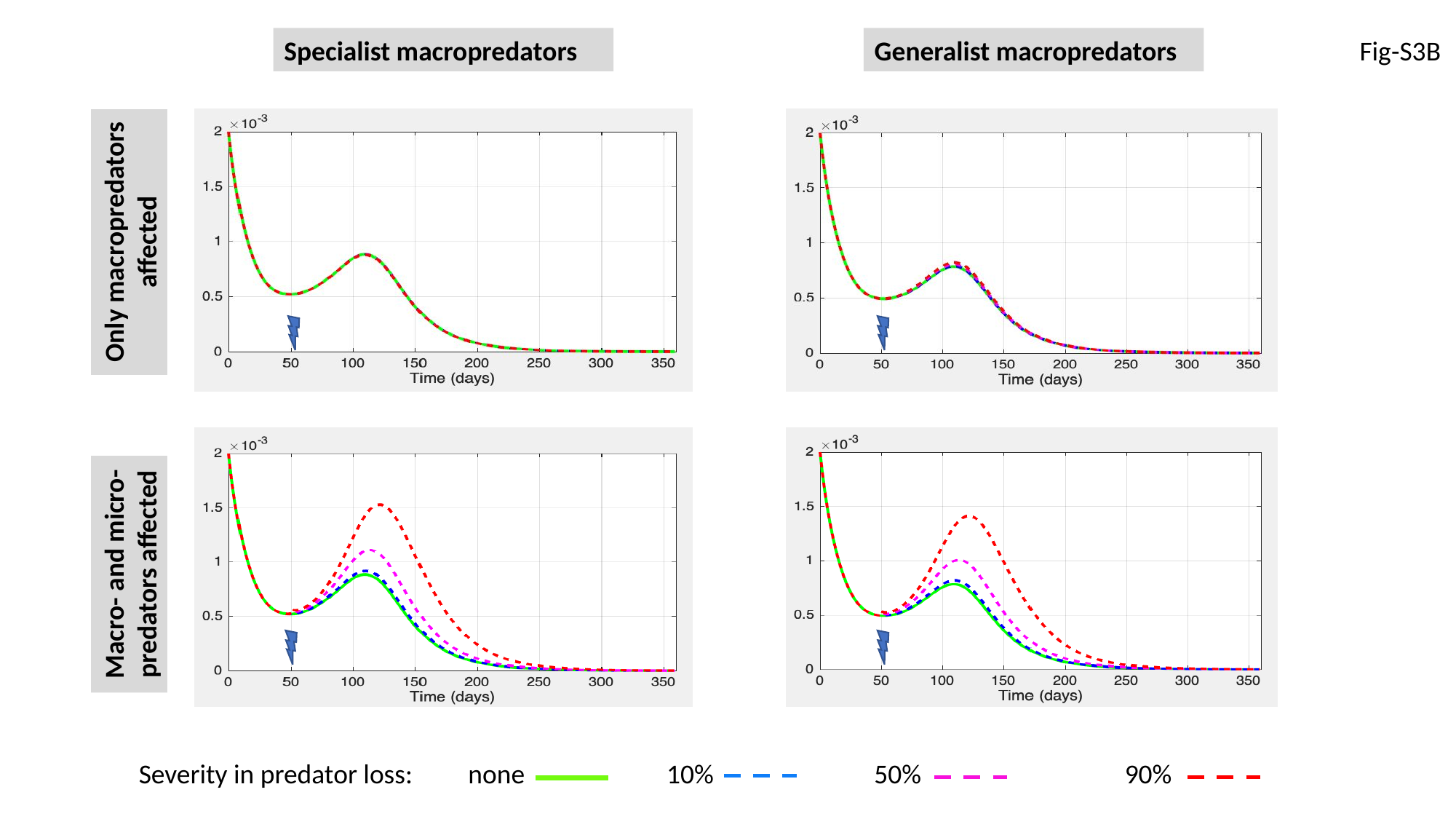

Specialist macropredators
Generalist macropredators
Fig-S3B
Only macropredators affected
Macro- and micro-
predators affected
Severity in predator loss: none 10% 50% 90%

## Slide 7
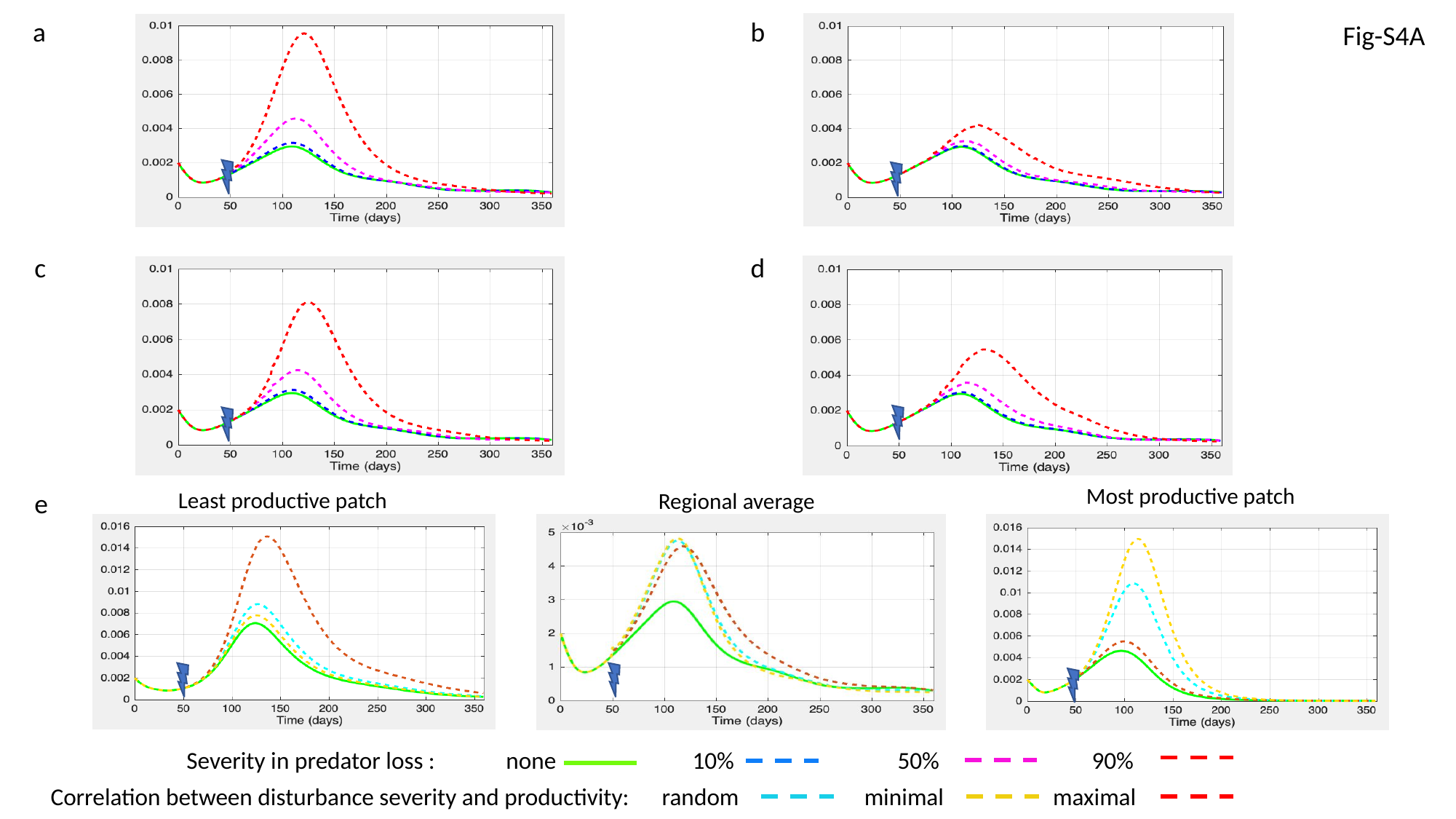

a
b
Fig-S4A
c
d
Most productive patch
Least productive patch
e
Regional average
Severity in predator loss : none 10% 50% 90%
Correlation between disturbance severity and productivity: random minimal maximal

## Slide 8
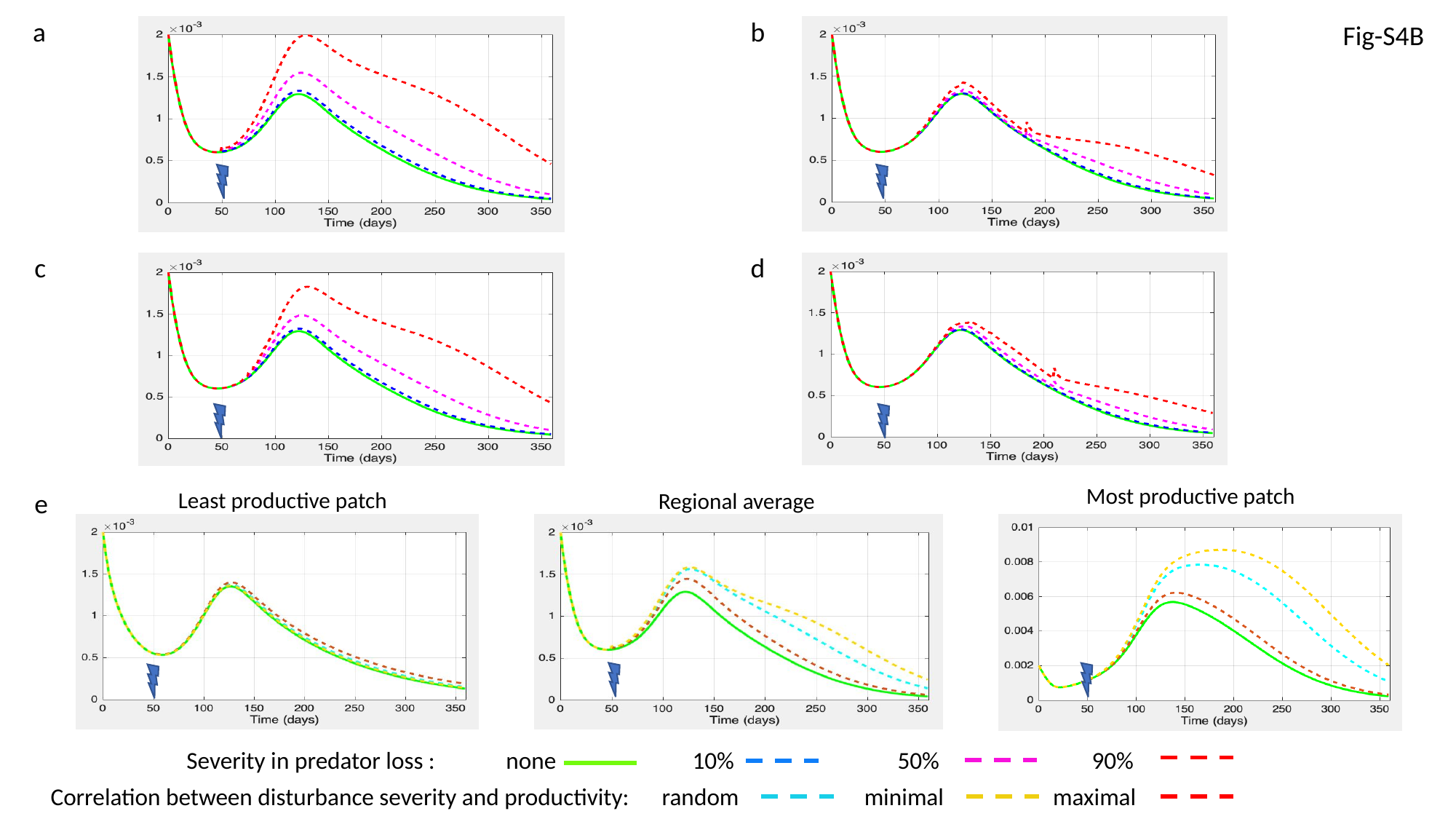

a
b
Fig-S4B
c
d
Most productive patch
Least productive patch
e
Regional average
Severity in predator loss : none 10% 50% 90%
Correlation between disturbance severity and productivity: random minimal maximal

## Slide 9
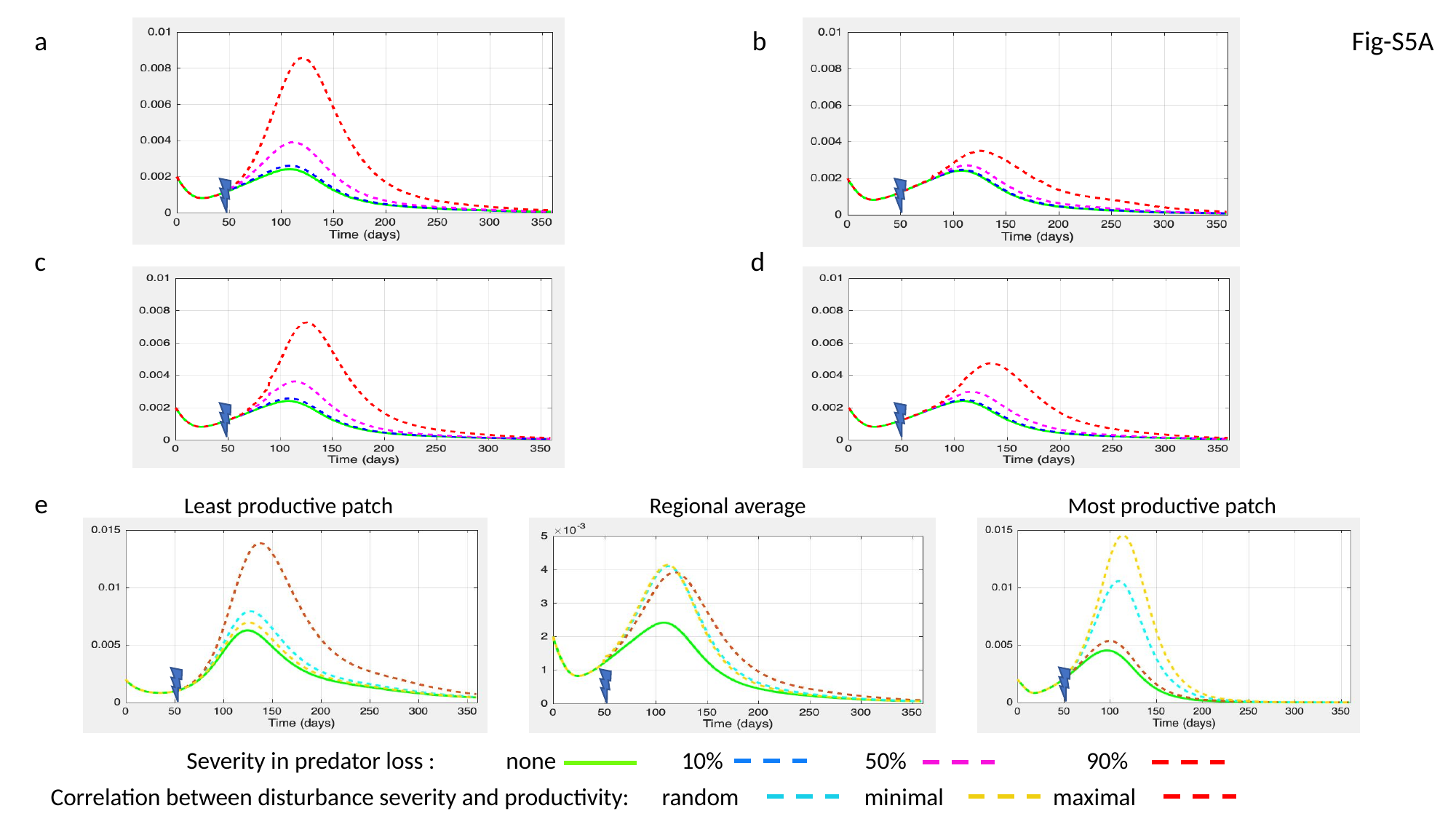

a
b
Fig-S5A
c
d
e
Least productive patch
Regional average
Most productive patch
Severity in predator loss : none 10% 50% 90%
Correlation between disturbance severity and productivity: random minimal maximal

## Slide 10
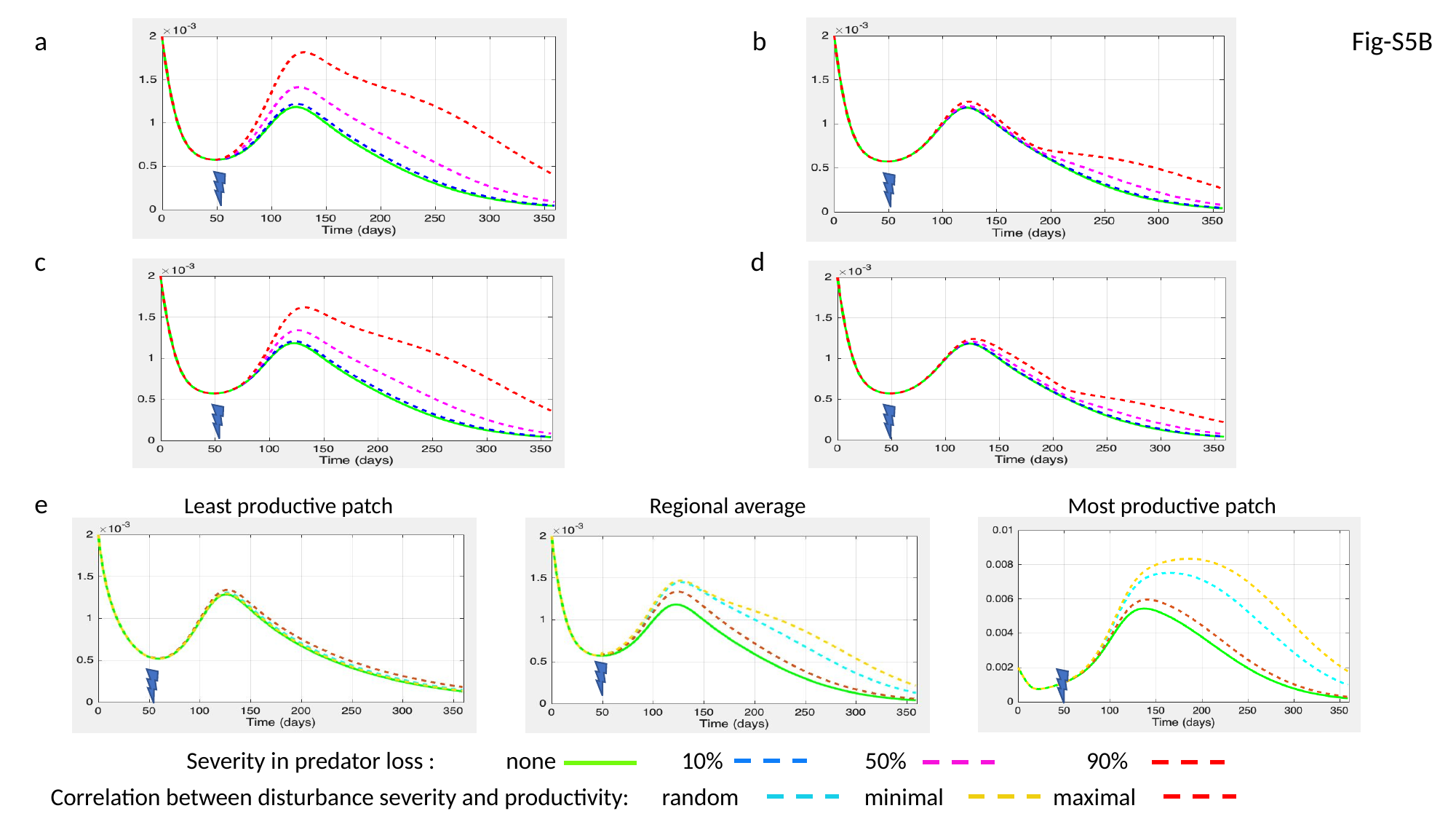

a
b
Fig-S5B
c
d
e
Least productive patch
Regional average
Most productive patch
Severity in predator loss : none 10% 50% 90%
Correlation between disturbance severity and productivity: random minimal maximal

## Slide 11
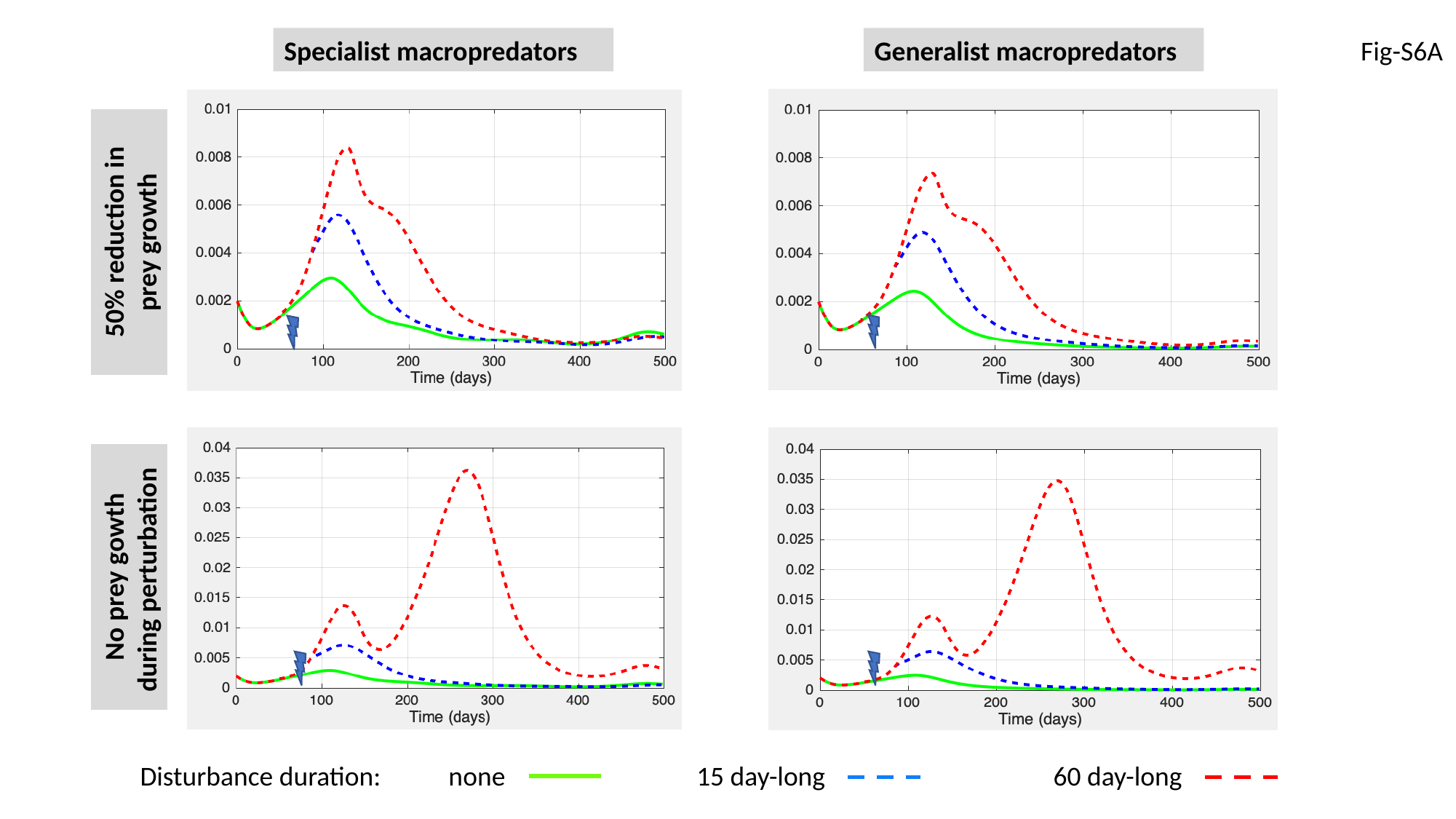

Specialist macropredators
Generalist macropredators
Fig-S6A
50% reduction in
prey growth
No prey gowth during perturbation
Disturbance duration: none 15 day-long 60 day-long

## Slide 12
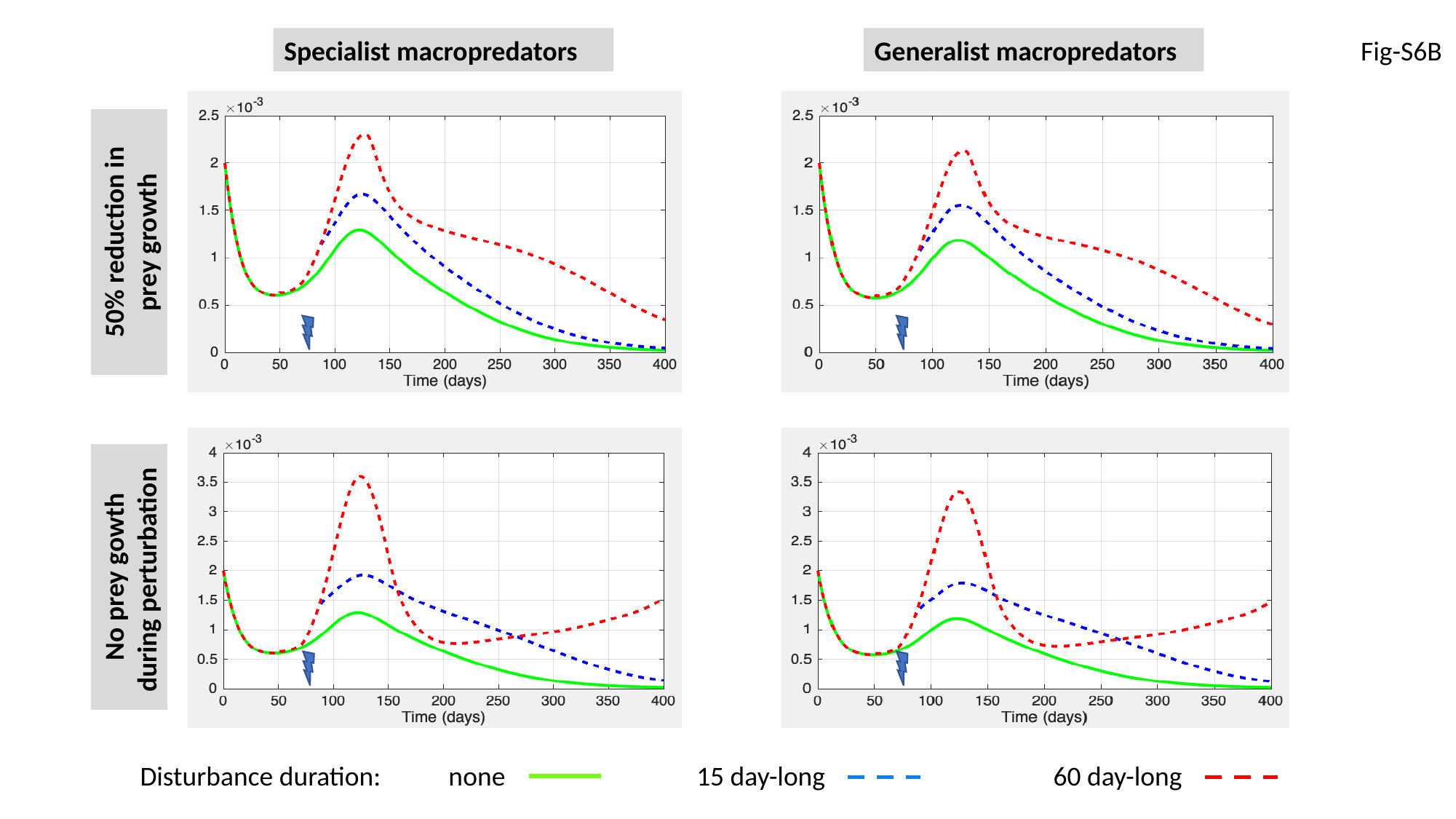

Specialist macropredators
Generalist macropredators
Fig-S6B
50% reduction in
prey growth
No prey gowth during perturbation
Disturbance duration: none 15 day-long 60 day-long
